# *O*-GlcNAcylation reprograms microglial inflammatory states and attenuates Alzheimer’s disease pathology

**DOI:** 10.1038/s41419-026-08862-3

**Published:** 2026-05-21

**Authors:** Dong Yeol Kim, Sang-Min Kim, Chanhaeng Lee, Inn-Oc Han

**Affiliations:** 1 Department of Physiology and Biophysics, College of Medicine, Inha University, Incheon, Korea; 2 Department of Biomedical Science, Program in Biomedical Science and Engineering, Inha University, Incheon, Korea

**Keywords:** Alzheimer's disease, Chronic inflammation, Neurodegeneration

## Abstract

Chronic neuroinflammation, primarily driven by microglia, is a hallmark and key contributor to Alzheimer’s disease (AD) progression. *O*-GlcNAcylation, a nutrient-sensitive post-translational modification, has emerged as a key regulator of cellular stress and inflammation, yet its role in microglial activation in AD remains unclear. We observed that hippocampal tissue from AD patients exhibits a marked reduction in *O*-GlcNAcylation, accompanied by enhanced pro-inflammatory M1 microglial polarization, elevated NF-κB signaling, and NLRP3 inflammasome activation. In an LPS-induced neuroinflammation model exhibiting AD-relevant inflammatory and cognitive features, as well as in in vitro microglial cultures, LPS exposure led to a pronounced decrease in *O*-GlcNAcylation, particularly within Iba1-positive microglia. Systemic or in vitro treatment with glucosamine (GlcN) effectively restored *O*-GlcNAc levels, suppressed M1-associated inflammatory pathways, and promoted an anti-inflammatory M2 phenotype. Mechanistically, GlcN enhanced *O*-GlcNAcylation of NF-κB subunits p65 and c-Rel, limiting their nuclear translocation and downstream pro-inflammatory gene expression. Notably, GlcN treatment ameliorated LPS-induced memory deficits and neuronal loss in mice. Collectively, these findings suggest that *O*-GlcNAcylation acts as a modulatory regulator of microglial activation and neuroinflammation in AD, and that enhancing *O*-GlcNAcylation may represent a potential therapeutic strategy to preserve immune homeostasis and neuronal integrity.

## Introduction

Alzheimer’s disease (AD) is a progressive neurodegenerative disorder and the leading cause of dementia worldwide, representing a growing socio-economic challenge with increasing life expectancy [1]. Clinically, AD is characterized by a gradual decline in memory, language, and executive function [2], while neuropathologically, it is defined by extracellular amyloid-β (Aβ) plaques and intracellular neurofibrillary tangles composed of hyperphosphorylated tau [3]. However, these canonical hallmarks alone do not fully account for the multifactorial and dynamic nature of disease progression [4]. Emerging evidence implicates chronic neuroinflammation, predominantly mediated by microglia, as a central driver of AD pathogenesis, critically shaping neuronal dysfunction and the trajectory of cognitive decline [5].

Microglia, the resident immune cells of the central nervous system (CNS), are central mediators of innate immune responses in the brain [6]. Under physiological conditions, microglia maintain tissue homeostasis through surveillance and synaptic pruning [7]. In AD, exposure to Aβ oligomers, tau aggregates, or neuronal debris triggers microglial activation, driving a phenotypic shift broadly categorized into pro-inflammatory M1 and anti-inflammatory M2 states [8–10]. M1 microglia secrete cytokines such as IL-1β, TNF-α, and IL-6, exacerbating synaptic dysfunction and neuronal injury, whereas M2 microglia facilitate Aβ clearance, resolve inflammation, and support tissue repair [11]. A sustained imbalance favoring M1 polarization, consistently observed in AD patient brains and experimental models, has been implicated as a central event in disease progression [12]. To recapitulate these processes, experimental models employing intracerebral administration of lipopolysaccharide (LPS), a Toll-like receptor 4 agonist, have been widely used [13]. LPS induces robust microglial activation, promotes pro-inflammatory M1 polarization, and activates the NF-κB pathway and NLRP3 inflammasome [14], leading to the maturation of IL-1β and IL-18 and ultimately triggering inflammatory cell death [15]. Importantly, LPS-induced neuroinflammation accelerates Aβ accumulation and tau hyperphosphorylation, which in turn further amplifies microglial activation and inflammatory signaling, establishing a vicious cycle that aggravates neuronal dysfunction and cell loss [16–19]. Within this pathological cascade, the microglial phenotypic shift toward chronic M1 activation represents the central driver of neurodegeneration, while NLRP3 inflammasome activation serves as an important mediator linking microglial dysfunction to neuronal injury, highlighting its potential as a therapeutic target in AD [20, 21].

Emerging evidence indicates that cellular metabolism critically shapes immune responses in the brain, with the hexosamine biosynthetic pathway (HBP) and its end-product, UDP-N-acetylglucosamine (UDP-GlcNAc), functioning as key metabolic checkpoints [22]. *O*-GlcNAcylation, the dynamic and reversible addition of *O-*GlcNAc to serine/threonine residues by *O*-GlcNAc transferase (OGT) and its removal by *O*-GlcNAcase (OGA), couples nutrient availability to cellular signaling, transcriptional regulation, and stress responses [22]. In the context of AD, reduced *O*-GlcNAcylation has been observed in affected brain regions and is linked to tau hyperphosphorylation and heightened microglial activation [23, 24]. However, the direct mechanistic role of *O*-GlcNAcylation in modulating microglial polarization—and the functional consequences of such regulation for neuroinflammation and neurodegeneration—remains poorly understood. In particular, whether enhancing *O*-GlcNAcylation can shift microglia from a deleterious M1 phenotype toward a neuroprotective M2 state in vivo has not been systematically addressed. Resolving this question is critical, as it could reveal a central metabolic-immune axis governing microglial function and offer new therapeutic avenues for AD.

Here, by integrating analyses of human AD brain samples with experimental models, we demonstrate that reduced *O*-GlcNAcylation is associated with aberrant microglial activation and pro-inflammatory signaling. Building on these observations, we provide the first comprehensive in vivo and in vitro evidence that pharmacological enhancement of *O*-GlcNAcylation directly modulates microglial polarization, suppresses NF-κB and NLRP3 activation, and protects against LPS-induced neuronal loss and cognitive deficits in an AD mouse model. By elucidating the crosstalk between metabolic signaling and innate immune regulation, our study uncovers a novel mechanism linking *O*-GlcNAcylation to microglial plasticity and identifies a promising therapeutic avenue to restore immune homeostasis and slow AD progression.

## Material and methods

### Cell culture

BV2 cells, an immortalized murine microglial cell line, were obtained from the Korean Cell Line Bank (Seoul, Korea). Cells were maintained in Dulbecco’s Modified Eagle’s Medium (DMEM; HyClone, UT, USA) supplemented with 5% fetal bovine serum (FBS; HyClone) and 100 U/mL penicillin-streptomycin (HyClone) under standard culture conditions. Cell line authentication was not performed. Cell lines were routinely screened for mycoplasma contamination to ensure experimental integrity and reproducibility.

### Human brain tissue samples

Human brain tissues were obtained from the Korea Brain Bank Network (KBBN), operating under the National Brain Bank Project funded by the Ministry of Science and ICT, South Korea. All procedures involving human tissues were approved by the Institutional Review Board of Inha University Hospital (approval number: 2023-05-018-000). The requirement for informed consent was waived by the Institutional Review Board due to the use of de-identified samples. Control brain samples included tissues from three donors with no significant AD pathology upon neuropathological assessment. AD brain samples comprised tissues from three donors with clinical and neuropathological confirmation of AD. The age range and cause of death for all human donors, along with detailed clinical and neuropathological information, are provided in Supplementary Table 1. All human brain tissues used in this study were obtained from the hippocampus. Quantitative analyses were specifically performed in the CA1 subregion, although the tissue blocks also contained adjacent hippocampal areas, including CA2, CA3, and the dentate gyrus. All methods were performed in accordance with the relevant guidelines and regulations.

### Whole protein lysate preparation

Human brain tissues, mouse hippocampal tissues, and cultured cells were homogenized in ice-cold RIPA buffer (Tech & Innovation, Chuncheon, Korea) supplemented with protease, phosphatase, and *O*-GlcNAcase inhibitors, including 1 mM PMSF, 1 mM DTT, 1 mg/mL aprotinin, 1 mg/mL leupeptin, 1 mM sodium orthovanadate, 1 mM sodium fluoride, and 1 mM streptozotocin. Homogenates were incubated on ice for 30 min with intermittent vortexing and subsequently centrifuged at 12,000 × *g* for 15 min at 4 °C to remove insoluble debris. The resulting supernatants, representing whole protein lysates, were collected, and protein concentrations were determined using the Bradford assay (Bio-Rad, Hercules, CA, USA).

### Cytoplasmic and nuclear fractionation

Harvested BV2 cells or mouse hippocampus tissues were homogenized in ice-cold cytoplasmic extraction buffer (10 mM HEPES, 1.5 mM MgCl₂, 200 mM sucrose, 0.5% NP-40, 10 mM KCl) supplemented with protease, phosphatase, and *O*-GlcNAcase inhibitors. Homogenates were incubated on ice for 1 h and subsequently centrifuged at 5000 rpm for 5 min at 4 °C to collect the cytoplasmic fraction (supernatant). The nuclear pellet was resuspended in nuclear extraction buffer (1 mM EDTA) and subjected to sonication (3–5 cycles of 5 s pulses with 30 s intervals on ice). Nuclear extracts were clarified by centrifugation at 12,000 × *g* for 10 min at 4 °C, and protein concentrations were determined using the Bradford assay (Bio-Rad).

### Wheat germ agglutinin (WGA) pull-down assay

Protein lysates (500 μg) derived from mouse hippocampus tissues or BV2 cells were incubated with pre-cleared WGA agarose beads (Vector Laboratories, USA) at 4 °C for 24 h with gentle rotation to enrich *O-*GlcNAc–modified glycoproteins. Prior to incubation, WGA beads were equilibrated and washed with phosphate-buffered saline (PBS) to remove preservatives and non-specifically bound proteins. Following incubation, beads were washed three times with ice-cold PBS to eliminate unbound proteins. Glycoproteins retained on the beads were eluted by boiling in SDS–PAGE sample buffer for 5 min and subsequently analyzed by SDS–PAGE and Western blotting using target-specific antibodies.

### Western blotting

Equal amounts of protein (30 μg) were resolved on 10% or 15% SDS–PAGE gels and transferred to nitrocellulose membranes (Cytiva, Marlborough, MA, USA). Membranes were blocked with 5% nonfat milk in PBST (PBS containing 0.1% Tween-20) for 1 h at room temperature and then incubated overnight at 4 °C with primary antibodies (1:1,000) targeting *O-*GlcNAc (sc-59623, RL2; Santa Cruz, Dallas, TX, USA), OGT (sc-74546, Santa Cruz), OGA (14771-1-AP, Proteintech, Rosemont, IL, USA), anti-amyloid β (sc-28365, Santa Cruz), β-actin (sc-47778, Santa Cruz), p-p65 (ab86229, Abcam, Cambridge, UK), p65 (sc-8008, Santa Cruz), p-IκB (sc-8404, Santa Cruz), IκB (sc-371, Santa Cruz), iNOS (610328, BD Biosciences, San Jose, CA, USA), COX-2 (A3560, Abclonal, Woburn, MA, USA), p-CREB (ab32096, Abcam), CREB (sc-377154, Santa Cruz), TLR4 (A0007, Abclonal), Arg1 (A4923, Abclonal), CD163 (A26411PM, Abclonal), CD86 (A16805, Abclonal), CD63 (A6554, Abclonal), c-Rel (sc-6955, Santa Cruz), α-tubulin (66031-1, Proteintech), p-STAT3 (9145, Cell Signaling Technology, Danvers, MA, USA), STAT3 (sc-8019, Santa Cruz), p-ERK (9101, Cell Signaling Technology), ERK (A16686, Abclonal), PPARγ (sc-7273, Santa Cruz), NLRP3 (sc-134306, Santa Cruz), ASC (sc-514414, Santa Cruz), TNF-α (11948, Cell Signaling Technology), IL-1β (12242, Cell Signaling Technology), cleaved caspase-1 (89332, Cell Signaling Technology), and caspase-1 (24232, Cell Signaling Technology). Following primary antibody incubation, membranes were washed three times with PBST and incubated with horseradish peroxidase-conjugated secondary antibodies (Invitrogen) for 1 h at room temperature. Protein bands were visualized using an enhanced chemiluminescence detection system (Bio-Rad) and, when required, quantified by densitometry using appropriate imaging software. Band intensities were quantified using ImageJ software. For each band, background intensity was measured from an adjacent area within the same lane and subtracted from the raw signal before normalization to the corresponding loading control.

### Immunohistochemistry and immunocytochemistry

BV2 cells cultured on poly-L-lysine-coated coverslips and 30-μm-thick mouse brain sections were washed with PBST (0.05% Triton X-100 in PBS) and blocked with 0.5% bovine serum albumin (BSA) in PBST for 2 h at room temperature. Samples were incubated overnight at 4 °C with primary antibodies diluted in blocking buffer, including anti-NeuN (ab24307, Abcam, Cambridge, UK), anti-*O*-GlcNAc (sc-59623, RL2, Santa Cruz), anti-PSD95 (ab18258, Abcam), anti-synaptophysin (ab8049, Abcam), anti-GFAP (G3893, Sigma-Aldrich, St. Louis, MO, USA), anti-Iba1 (178846, Abcam), anti-CD163 (A26411PM, Abclonal), anti-CD86 (A16805, Abclonal), and anti-amyloid β (sc-28365, Santa Cruz) (all 1:100). After washing, samples were incubated with Alexa Fluor-conjugated secondary antibodies (Invitrogen) for 1 h at room temperature in the dark. Nuclei were counterstained with DAPI (Invitrogen). For Iba1 immunohistochemistry, brain sections were incubated with biotinylated secondary antibodies and visualized using diaminobenzidine (DAB; Vector Laboratories). DAB-stained sections were dehydrated and imaged by bright-field microscopy (Olympus, Tokyo, Japan). Fluorescence images were acquired using a Zeiss LSM 980 confocal microscope (Zeiss, Oberkochen, Germany). Quantitative image analysis, including fluorescence intensity and cell morphology, was performed using ImageJ (NIH) and ZEN software (Zeiss).

### Golgi–Cox staining

Golgi–Cox staining was performed using the FD Rapid GolgiStain™ Kit (FD NeuroTechnologies, Columbia, MD, USA) according to the manufacturer’s protocol with minor modifications. Following transcardial perfusion, mouse brains were rapidly removed, rinsed in ice-cold 0.1 M PBS, and immediately immersed in the impregnation solution. Samples were kept at room temperature in the dark for 2 weeks, with the solution replaced after the first 24 h. Subsequently, tissues were transferred to Solution C and incubated for 48–72 h at 4 °C. Coronal sections (100 µm) were obtained using a vibratome (Leica Microsystems, Wetzlar, Germany) and mounted on gelatin-coated slides. After air-drying at room temperature, sections were developed through sequential immersion in developer solutions, rinsed in distilled water, dehydrated in graded ethanol, cleared in xylene, and coverslipped with a non-aqueous mounting medium (Sigma-Aldrich). Golgi-stained neurons were visualized by bright-field microscopy (Olympus, Tokyo, Japan). For dendritic spine analysis, pyramidal neurons were selected from the CA1 region of the hippocampus. Spine density was quantified in apical and basal dendrites located in the stratum radiatum. Only well-impregnated and clearly isolated neurons were included for quantification.

### Experimental animals and drug administration

All animal procedures were approved by the Institutional Animal Care and Use Committee of Inha University (Approval Number: 190920-655) and conducted in accordance with institutional guidelines for the care and use of laboratory animals. Male C57BL/6J mice (7 weeks old; DBL, Chungbuk, Korea) were maintained under controlled conditions with a 12-h light/dark cycle, ambient temperature of 22 ± 2 °C, and *ad libitum* access to food and water. Mice were acclimated for one week prior to experimental procedures. Animals were randomly assigned to experimental groups using a random number generator. To induce neuroinflammation and hippocampal neuron degeneration, lipopolysaccharide (LPS; 15 μg in 2 μL sterile saline; Sigma-Aldrich) was stereotaxically injected into the lateral ventricle using the following coordinates relative to bregma: anterior-posterior, −0.3 mm; mediolateral, +1.0 mm; dorsoventral, −2.5 mm from the brain surface. Injections were performed with a 26-gauge stainless steel needle attached to a 10 μL Hamilton microsyringe at a slow, controlled rate. Following injection, the needle was left in place for 10 min to prevent backflow before gradual withdrawal. For pharmacological modulation, glucosamine (GlcN; 200 mg/kg; Sigma-Aldrich) was administered via intraperitoneal injection three times per week for four weeks, beginning one day after LPS administration. Control animals received equivalent volumes of vehicle (sterile saline). Body weight and general health were monitored throughout the experimental period to ensure well-being and reproducibility of results.

### Behavioral assessments

All behavioral apparatuses were thoroughly cleaned with 70% ethanol between trials to eliminate residual olfactory cues and ensure unbiased performance. Behavioral tests were conducted during the light phase (09:00–17:00 h) under consistent lighting and noise-controlled conditions. Animals were habituated to the testing room for at least 30 min prior to each experiment to minimize stress-related variability. Experimental procedures were randomized, and investigators were blinded to treatment groups to reduce potential bias.

#### Passive avoidance test

The passive avoidance test was conducted using a two-compartment shuttle box consisting of an illuminated chamber and a dark chamber separated by a guillotine door (Jeungdo Bio & Plant Co., Ltd., Seoul, Korea). During the habituation phase, mice were placed in the apparatus and allowed to freely explore both compartments for 10 min. On the training day, mice were initially placed in the illuminated chamber and permitted to explore for 10 s before the door to the dark chamber was opened. Upon full entry into the dark compartment, the door was closed, and a single mild foot shock (0.5 mA, 1 s) was delivered via the grid floor. Mice were then returned to their home cages. Each mouse underwent three training trials separated by 1-h intervals. Memory retention was assessed 24 h after training by reintroducing the mice into the illuminated chamber and recording the latency to enter the dark compartment (maximum cutoff time: 300 s) in the absence of foot shock. Increased latency was interpreted as improved aversive memory retention. All procedures were performed under standardized lighting and noise conditions, and investigators were blinded to treatment groups to minimize bias.

#### Novel object test

The novel object recognition test was conducted in an open-field arena (40 × 40 × 40 cm) under low ambient light to minimize stress and visual distraction. On day 1 (habituation), mice were allowed to freely explore the empty arena for 10 min. On day 2 (familiarization phase), mice were placed in the arena containing two identical objects and allowed to explore for 10 min. Twenty-four hours later, during the test phase, one familiar object was replaced with a novel object differing in shape and texture, and mice were allowed to explore for 5 min. Exploration was defined as directing the nose toward the object within ≤2 cm or making contact with the object using the vibrissae. The time spent exploring each object was recorded, and a discrimination index was calculated as follows: (time spent on novel object − time spent on familiar object)/(time spent on novel object + time spent on familiar object). Higher values of the discrimination index indicate superior recognition memory. All procedures were conducted under consistent lighting and noise conditions, and experimenters were blinded to treatment groups to minimize bias.

#### Morris water maze test

Spatial learning and memory were assessed using the Morris water maze test in a circular pool (90 cm diameter) filled with water maintained at 23–25 °C. The pool was conceptually divided into four quadrants, and a hidden platform (1–2 cm below the water surface) was positioned in a target quadrant. A camera mounted above the tank recorded swimming trajectories for subsequent analysis. During training, each mouse was placed in the water facing the tank wall from a different quadrant in each trial to prevent quadrant bias. Mice underwent three training trials per day over five consecutive days to assess the acquisition of spatial memory. On day 6, memory retention was evaluated using a probe trial in which the platform was removed, and mice were allowed to swim freely. Escape latency during training trials and swimming paths during both training and probe trials were recorded and analyzed offline using Smart 3.0 software (Panlab, S.L.U, Barcelona, Spain). All experiments were conducted under consistent lighting and noise conditions, and experimenters were blinded to treatment groups. Water was refreshed between trials to eliminate olfactory cues, and mice were gently dried and returned to their home cages after each session to minimize stress.

### Statistical analyses

All quantitative data are presented as mean ± standard error of the mean (SEM) from at least three independent experiments, with sample sizes indicated in the figure legends. No statistical methods were used to predetermine sample size. Sample sizes were based on prior experience with experimental models and consistency with previously published studies. No samples or animals were excluded from the analysis. No blinding was performed during the experiments or outcome assessment. Normality of data distribution was assessed prior to statistical testing. Variance was assumed to be similar between groups. Comparisons between two groups were performed using unpaired two-tailed Student’s *t*-tests. For comparisons involving three or more groups, one-way analysis of variance (ANOVA) followed by Tukey’s post hoc multiple comparisons test was applied. Statistical analyses were conducted using GraphPad Prism 10.0 (GraphPad Software, San Diego, CA, USA). A *p*-value of less than 0.05 was considered statistically significant.

## Results

### Reduced O-GlcNAcylation accompanies neuroinflammation and pro-inflammatory microglial activation in the hippocampus of AD patients

**Fig. 1:**
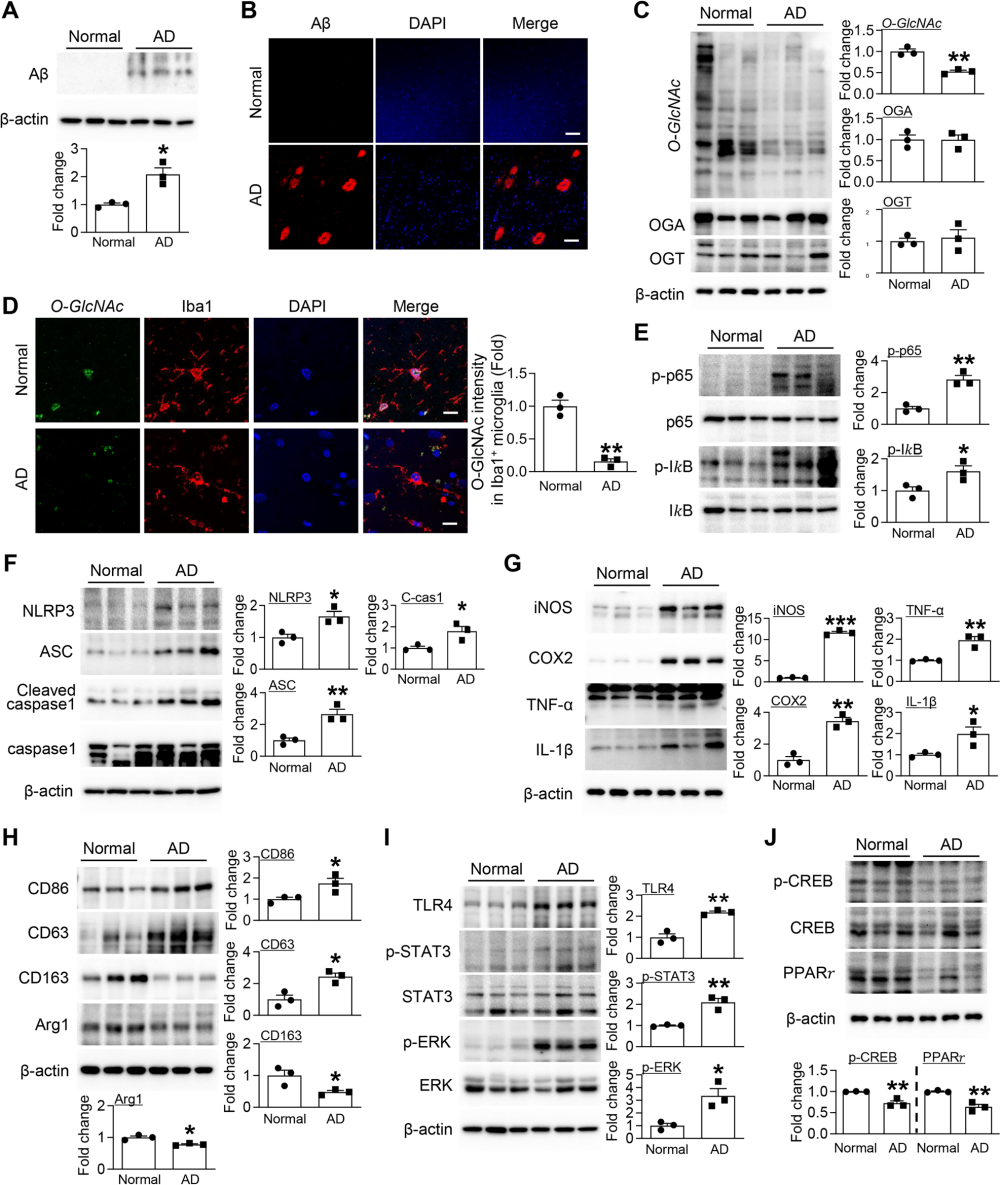
Reduced *O*-GlcNAcylation accompanies neuroinflammation and pro-inflammatory microglial activation in the hippocampus of AD patients. **A** Western blot analysis of hippocampus tissue lysates was performed using Aβ and β-actin antibodies. Quantification of Aβ expression was normalized to β-actin (*n* = 3 per group). **B** Representative immunohistochemical images of Aβ in human hippocampus sections. Scale bar = 100 μm (*n* = 3 per group). **C** Western blot analysis of *O*-GlcNAcylation levels and the expression of OGT, OGA, and β-actin in hippocampus lysates. Quantification was normalized to β-actin (*n* = 3 per group). **D** Representative immunofluorescent images of microglia stained for *O-GlcNAc* (green), Iba1 (red), and DAPI (blue). Scale bar = 10 μm (*n* = 3 per group). **E** Western blot analysis of NF-κB signaling components in hippocampus lysates, including phospho-p65 (p-p65), total p65, phospho-IκB (p-IκB), and total IκB. Quantification of p-p65 was normalized to p65; p-IκB was normalized to IκB (*n* = 3 per group). **F** Western blot analysis of inflammasome-related proteins in hippocampus lysates, including NLRP3, ASC, cleaved caspase-1, total caspase-1, and β-actin. Quantification of NLRP3 and ASC was normalized to β-actin; cleaved caspase-1 was normalized to total caspase-1 (*n* = 3 per group). **G** Western blot analysis of neuroinflammatory markers in hippocampus lysates, including iNOS, COX-2, TNF-α, IL-1β, and β-actin. Quantification was normalized to β-actin (*n* = 3 per group). **H** Western blot analysis of microglia polarization markers in hippocampus lysates, including CD86, CD68, CD163, Arg1, and β-actin. Quantification was normalized to β-actin (*n* = 3 per group). **I** Western blot analysis of TLR4, phosphorylated and total STAT3, and ERK in hippocampus lysates. Quantification of p-STAT3 was normalized to STAT3; p-ERK was normalized to ERK; TLR4 was normalized to β-actin (*n* = 3 per group). **J** Western blot analysis of PPARγ, phosphorylated CREB (p-CREB), total CREB, and β-actin in hippocampus lysates. Quantification of p-CREB was normalized to total CREB; PPARγ was normalized to β-actin (*n* = 3 per group). Data are presented as mean SEM; *p < 0.05, **p < 0.01, ***p < 0.001. Statistical analysis was performed using the Student *t*-test.

Postmortem analysis of the hippocampus from AD patients revealed hallmark neuropathological features of neurodegeneration, including pronounced Aβ accumulation (Fig. 1A, B). Concomitant with these pathological alterations, global protein *O*-GlcNAcylation was significantly reduced in AD hippocampal tissue compared to age-matched control brains, suggesting that impaired *O*-GlcNAcylation may contribute to the initiation or amplification of disease-associated cellular stress (Fig. 1C). To investigate whether this reduction preferentially affects microglia, immunofluorescence staining was performed. Microglia in the AD hippocampus exhibited significantly lower *O*-GlcNAcylation levels compared to surrounding neurons and non-microglial cells, indicating a cell-type-specific vulnerability to *O-*GlcNAc depletion (Fig. 1D). The selective reduction of *O-*GlcNAc in microglia was accompanied by pronounced activation of pro-inflammatory signaling pathways, including NF-κB, as evidenced by elevated phosphorylation of p65 and IκB (Fig. 1E). Components of the NLRP3 inflammasome, including NLRP3, ASC, and cleaved caspase-1, were upregulated, consistent with inflammasome assembly and heightened neuroinflammatory responses (Fig. 1F). In line with these molecular changes, pro-inflammatory mediators, such as iNOS, COX-2, TNF-α, and IL-1β, were significantly increased (Fig. 1G). Phenotypic analysis of microglia further revealed a shift toward a pro-inflammatory M1-like state. M1-associated markers, including CD86 and CD68, were elevated, whereas M2-associated markers, such as CD163 and Arg1, were markedly downregulated (Fig. 1H). Correspondingly, signaling pathways promoting M1 polarization, including phosphorylated ERK, STAT3, and TLR4 activation, were upregulated, while M2-associated regulators, such as PPARγ and phosphorylated CREB, were diminished in AD hippocampal tissue (Fig. 1I, J). Collectively, these data suggest that the observed reduction in global *O*-GlcNAcylation may preferentially influence microglia, thereby facilitating a shift toward a pro-inflammatory M1 phenotype and potentially activating downstream neuroinflammatory pathways.

### LPS reduced O-GlcNAcylation, and GlcN restores LPS-induced O-GlcNAcylation in microglia of the LPS-induced neuroinflammation model relevant to AD and in vitro microglial cells

**Fig. 2:**
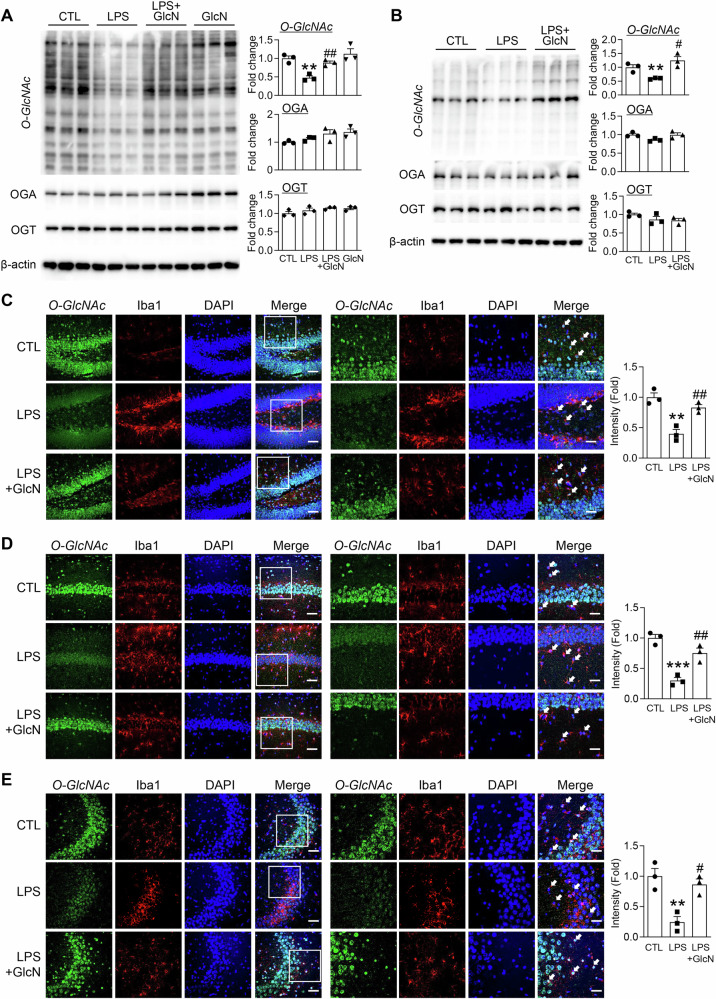
LPS reduced *O*-GlcNAcylation, and GlcN restores LPS-induced *O*-GlcNAcylation in the microglia of LPS-induced AD-relevant inflammatory mice and in vitro microglial cells. **A**, **C**–**E** Mice received lateral ventricle injections of LPS (15 μg), and selected groups were treated with GlcN (200 mg/kg, intraperitoneally, three times per week for 4 weeks). **A** Western blot analysis of hippocampus lysates was performed using antibodies against *O-GlcNAc*, OGT, OGA, and β-actin. Quantification of *O-GlcNAc*, OGT, and OGA was normalized to β-actin (*n* = 3 per group). **B** BV2 cells were treated with LPS (400 ng/mL) in the presence or absence of GlcN (5 mM) for 24 h. Western blot analysis was performed for *O-GlcNAc*, OGT, OGA, and β-actin, with quantification normalized to β-actin (*n* = 3 per group). **C**–**E** Representative immunofluorescence images of the hippocampus showing *O-GlcNAc* (green), Iba1 (red), DAPI (blue), and merged channels. Scale bar = 50 μm. Enlarged regions are outlined with white squares. Scale bar = 10 μm (*n* = 3 per group). Data are presented as mean SEM; *p < 0.05, **p < 0.01, ***p < 0.001 versus control, ^#^*p* < 0.05, ^##^*p* < 0.01, ^###^*p* < 0.001 versus LPS. Statistical analysis was performed using one-way ANOVA with Tukey’s post hoc multiple comparison test.

To investigate the link between reduced *O*-GlcNAcylation, neuroinflammation, and AD pathology observed in human brains, we examined an LPS-induced neuroinflammation mouse model relevant to AD. Stereotaxic administration of LPS into the hippocampus elicited a pronounced decrease in global protein *O*-GlcNAcylation (Fig. 2A), consistent with previous reports that inflammatory stimuli disrupt *O*-GlcNAc homeostasis [25, 26]. Systemic treatment with GlcN effectively restored total *O-*GlcNAc levels in the hippocampus to near-baseline values (Fig. 2A). Immunofluorescence analysis further revealed that the LPS-induced decrease in *O*-GlcNAcylation was particularly pronounced within Iba1-positive microglia, highlighting a cell-type-specific vulnerability of microglial *O-*GlcNAc homeostasis to inflammatory challenge. Importantly, GlcN treatment reversed this reduction, reinstating *O-*GlcNAc levels specifically in microglia (Fig. 2C–E). To confirm these effects at the cellular level, BV2 microglial cells were exposed to LPS in vitro with or without GlcN supplementation. Western blot analysis demonstrated that LPS significantly decreased *O*-GlcNAcylation in BV2 cells, whereas co-treatment with GlcN restored *O-*GlcNAc levels to baseline (Fig. 2B). Collectively, these findings indicate that GlcN effectively counteracts inflammation-induced depletion of *O*-GlcNAcylation in microglia, both in vivo in the hippocampus and in vitro in cultured microglial cells.

### GlcN-mediated O-GlcNAcylation augmentation attenuates neuronal damage in an LPS-induced neuroinflammation model relevant to AD

**Fig. 3:**
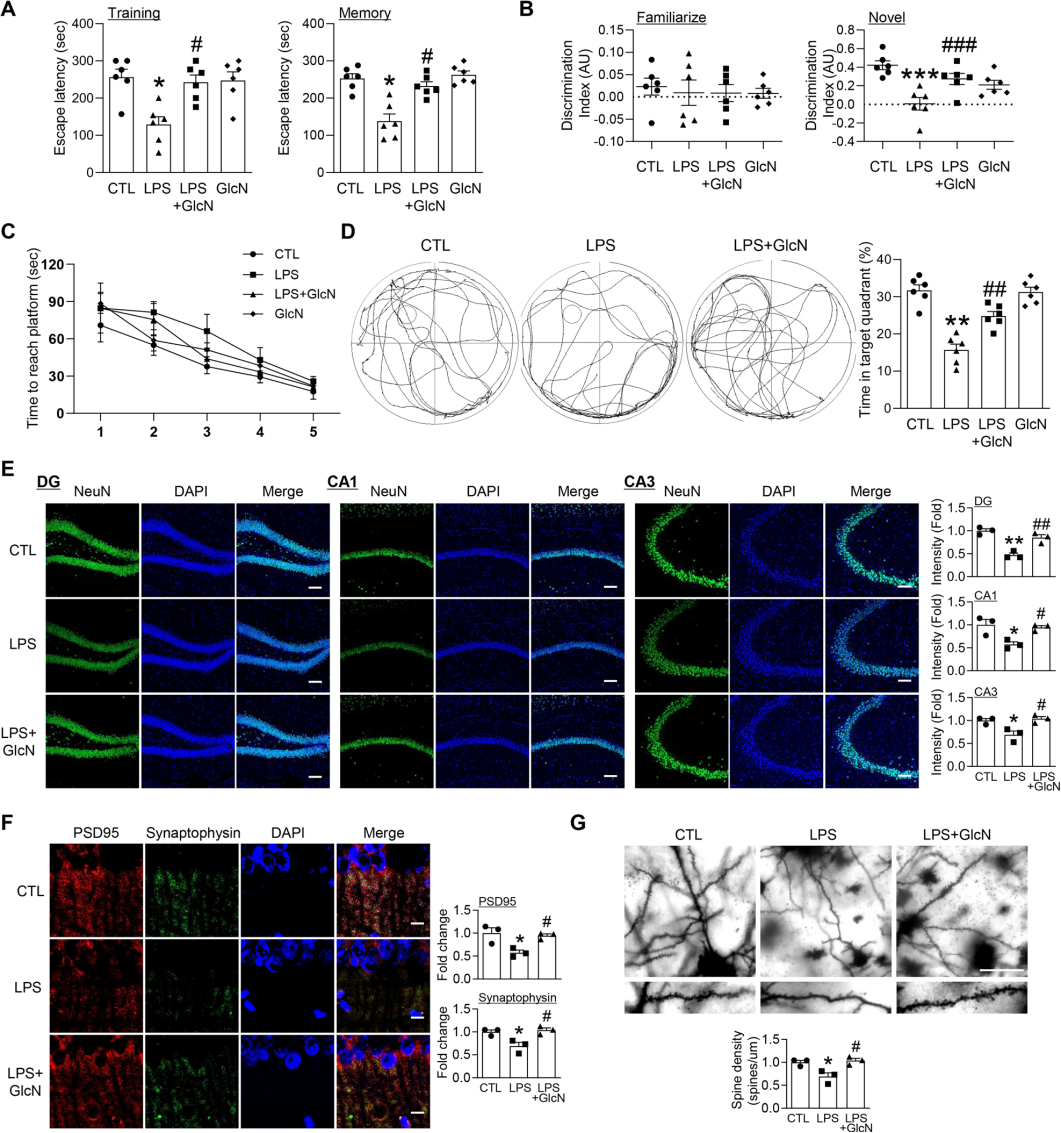
GlcN-mediated *O*-GlcNAcylation augmentation attenuates neuronal damage in an LPS-induced AD-relevant neuroinflammatory mouse model. Mice received intracerebroventricular (ICV) injections of LPS (15 μg), and GlcN was administered intraperitoneally at 200 mg/kg, three times per week for 4 weeks. **A** Passive avoidance test: mice were initially placed in the illuminated chamber and received a mild foot shock upon entering the dark compartment. Memory retention was evaluated 24 h later by measuring latency to re-enter the dark chamber (*n* = 6 per group). **B** Novel object recognition test: mice were familiarized with two identical objects, and during the test session, one familiar object was replaced with a novel object. Time spent exploring the novel versus familiar object was recorded as an index of recognition memory (*n* = 6 per group). **C** Morris water maze: spatial learning was assessed over multiple trials as mice located a hidden platform using distal visual cues; escape latency across training days was recorded (*n* = 6 per group). **D** Probe trial: 24 h after the final Morris water maze training, the platform was removed, and time spent in the target quadrant was measured as an index of spatial memory retention (*n* = 6 per group). **E** Representative immunofluorescence images of hippocampal NeuN (green) and DAPI (blue). Scale bar = 100 μm (*n* = 3 per group). **F** Representative immunofluorescence images of hippocampal PSD95 (red), synaptophysin (green), and DAPI (blue). Scale bar = 10 μm (*n* = 3 per group). **G** Representative images of dendritic morphology in hippocampal CA1 visualized by Golgi staining. Scale bar = 10 μm (*n* = 3 per group). Data are presented as mean SEM; *p < 0.05, **p < 0.01, ***p < 0.001 versus control, ^#^*p* < 0.05, ^##^*p* < 0.01, ^###^*p* < 0.001 versus LPS. Statistical analysis was performed using one-way ANOVA with Tukey’s post hoc multiple comparison test.

We next examined whether the enhancement of *O*-GlcNAcylation might mitigate key pathological features of AD. In the passive avoidance paradigm, LPS-challenged mice exhibited a marked reduction in step-through latency compared to vehicle-treated controls, indicative of impaired contextual memory retrieval. Notably, GlcN treatment significantly restored avoidance behavior, suggesting a protective effect on hippocampus-dependent memory consolidation (Fig. 3A). Similarly, in the novel object recognition test, LPS-treated animals demonstrated a significant decrease in the discrimination index, reflecting deficits in recognition memory. GlcN administration effectively rescued this impairment, indicating that enhancing *O*-GlcNAcylation mitigates LPS-induced cognitive deficits, likely via modulation of neuroinflammatory pathways (Fig. 3B). In the Morris water maze, escape latencies during acquisition training were comparable across all groups, indicating that spatial learning acquisition was not significantly affected by LPS or GlcN treatment (Fig. 3C). However, in the probe trial, LPS-injected mice spent significantly less time in the target quadrant relative to controls, indicative of impaired spatial memory retention. GlcN treatment restored target quadrant performance, further supporting its beneficial role in preserving hippocampus-dependent memory function (Fig. 3D). To investigate the neurobiological basis underlying the observed behavioral improvements, we examined hippocampal neuronal integrity using NeuN immunofluorescence across key subregions. LPS administration induced a pronounced reduction in NeuN-positive cell density within the dentate gyrus (DG), CA1, and CA3 regions, consistent with widespread neuronal loss or dysfunction under neuroinflammatory conditions. Importantly, systemic GlcN treatment significantly preserved NeuN immunoreactivity across these subregions, indicating a robust neuroprotective effect (Fig. 3E) that parallels the improvements observed in hippocampus-dependent memory tasks. Synaptic integrity was then assessed by immunostaining for postsynaptic density protein PSD95 and presynaptic vesicle marker synaptophysin in the CA1 region. LPS exposure led to marked reductions in both markers, indicating synaptic degeneration, whereas GlcN administration effectively restored PSD95 and synaptophysin density to near-control levels (Fig. 3F). Complementary Golgi–Cox staining demonstrated that LPS treatment markedly reduced dendritic spine density in apical dendrites of CA1 pyramidal neurons, with a comparable reduction observed in basal dendrites. These LPS-induced alterations were significantly rescued or partially restored by GlcN treatment (Fig. 3G and Fig. S1). These findings indicate that GlcN-mediated enhancement of *O*-GlcNAcylation preserves neuronal and synaptic integrity, providing a structural basis for improved hippocampal memory and reduced neuroinflammation in an LPS-induced model that captures key inflammatory and cognitive features relevant to AD.

### GlcN suppresses neuroinflammatory pathways and glial activation induced by LPS

**Fig. 4:**
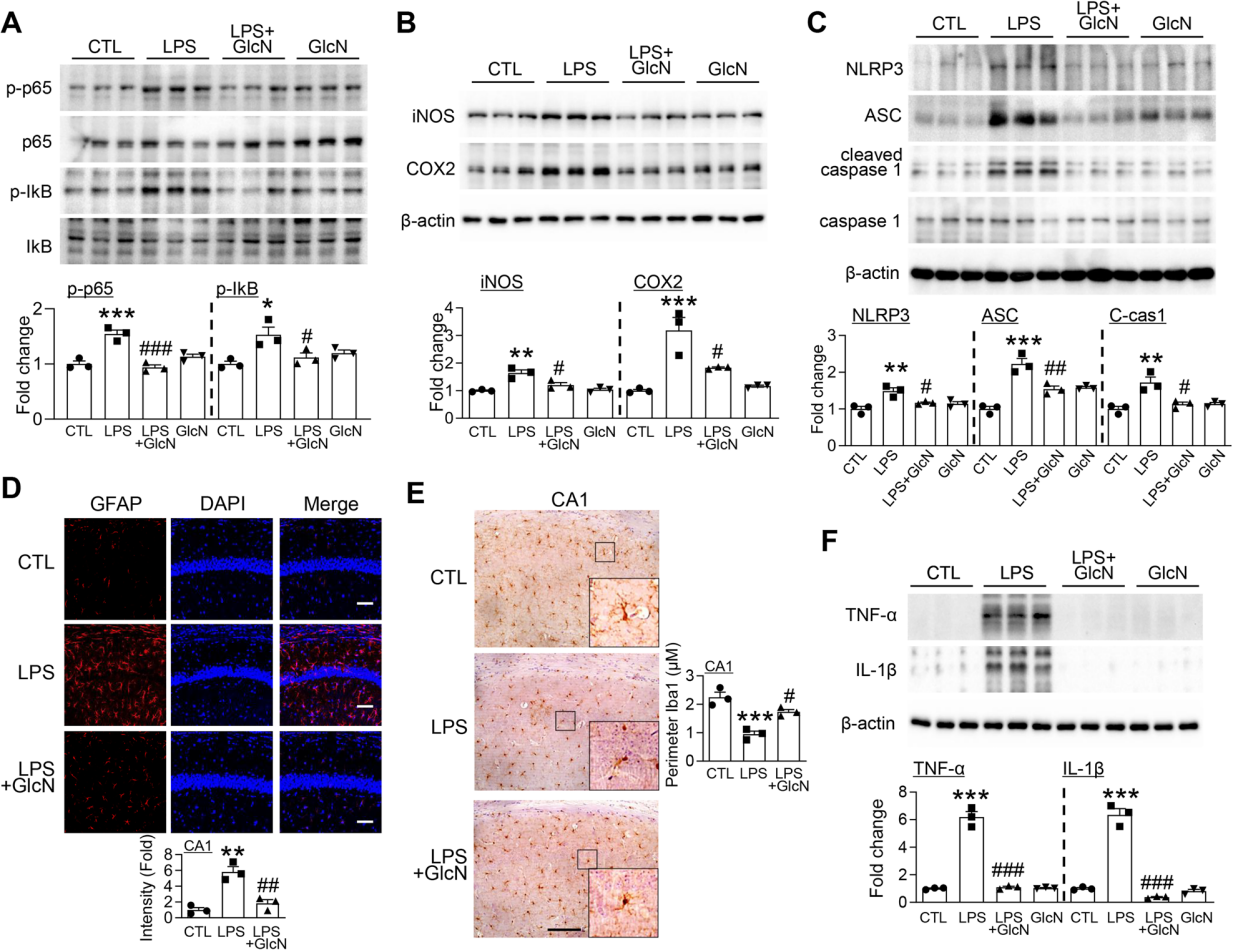
GlcN suppresses neuroinflammatory pathways and glial activation induced by LPS. Mice received ICV injections of LPS (15 μg) into the lateral ventricle, and GlcN was administered intraperitoneally at 200 mg/kg, three times per week for 4 weeks. **A** Western blot analysis of NF-κB pathway components, including phospho-p65 (p-p65), phospho-IκB (p-IκB), total p65, and total IκB in hippocampus lysates. Quantification of p-p65 and p-IκB was normalized to total p65 and total IκB, respectively (*n* = 3 per group). **B** Western blot analysis of inflammatory markers iNOS and COX-2 in hippocampus lysates. Protein levels were normalized to β-actin (*n* = 3 per group). **C** Western blot analysis of inflammasome-related proteins NLRP3, ASC, cleaved caspase-1, and total caspase-1. NLRP3 and ASC levels were normalized to β-actin, and cleaved caspase-1 was normalized to total caspase-1 (*n* = 3 per group). **D** Representative immunofluorescence images of the hippocampus showing GFAP (red) and DAPI (blue). Scale bar = 50 μm (*n* = 3 per group). **E** Iba1 immunohistochemistry to assess microglial morphology in the hippocampus. Scale bar = 100 μm (*n* = 3 per group). **F** Western blot analysis of inflammatory cytokines TNF-α and IL-1β in hippocampus lysates. Protein levels were normalized to β-actin (*n* = 3 per group). Data are presented as mean SEM; *p < 0.05, **p < 0.01, ***p < 0.001 versus control, ^#^*p* < 0.05, ^##^*p* < 0.01, ^###^*p* < 0.001 versus LPS. Statistical analysis was performed using one-way ANOVA with Tukey’s post hoc multiple comparison test.

To elucidate the molecular mechanisms underlying LPS-induced neuroinflammation and the potential anti-inflammatory effects of GlcN, we analyzed key components of canonical NF-κB signaling and downstream inflammatory mediators. Western blot analysis revealed that LPS administration robustly increased the phosphorylation of p65 and IκB, indicating activation of NF-κB signaling (Fig. 4A). This was accompanied by marked upregulation of inducible nitric oxide synthase (iNOS) and cyclooxygenase-2 (COX-2), canonical pro-inflammatory effectors. Notably, systemic GlcN treatment significantly attenuated the phosphorylation of NF-κB components and reduced the expression of iNOS and COX-2 (Fig. 4A, B), demonstrating effective suppression of NF-κB–mediated inflammatory signaling. Given the pivotal role of the NLRP3 inflammasome in microglia-driven neuroinflammation, we next examined the expression of NLRP3, ASC, and cleaved caspase-1. LPS challenge induced pronounced upregulation of all three components, whereas GlcN markedly diminished their expression, suggesting that GlcN also mitigates inflammasome activation and downstream pro-inflammatory cascades (Fig. 4C). To assess glial activation at the cellular level, we performed immunofluorescence for GFAP and Iba1. LPS exposure elicited pronounced astrocyte reactivity, reflected by increased GFAP immunoreactivity and hypertrophic morphology (Fig. 4D and Fig. S2A). Microglia similarly adopted an activated ameboid phenotype with enlarged soma and retracted processes (Fig. 4E and Fig. S2B). Importantly, GlcN administration attenuated both astrocytic and microglial activation, as evidenced by reduced GFAP expression and a shift of microglia toward a ramified, surveillant morphology (Fig. 4D, E and Fig. S2A, B). Complementary analysis of pro-inflammatory cytokines revealed that LPS strongly upregulated TNF-α and IL-1β expression in the hippocampus, consistent with activation of NF-κB and inflammasome pathways. GlcN treatment significantly suppressed the levels of these cytokines (Fig. 4F), further confirming its anti-inflammatory efficacy. Collectively, these findings indicate that GlcN effectively suppresses LPS-induced neuroinflammation at multiple levels.

### Restoration of O-GlcNAcylation in microglia attenuates LPS-induced inflammatory and inflammasome signaling

**Fig. 5:**
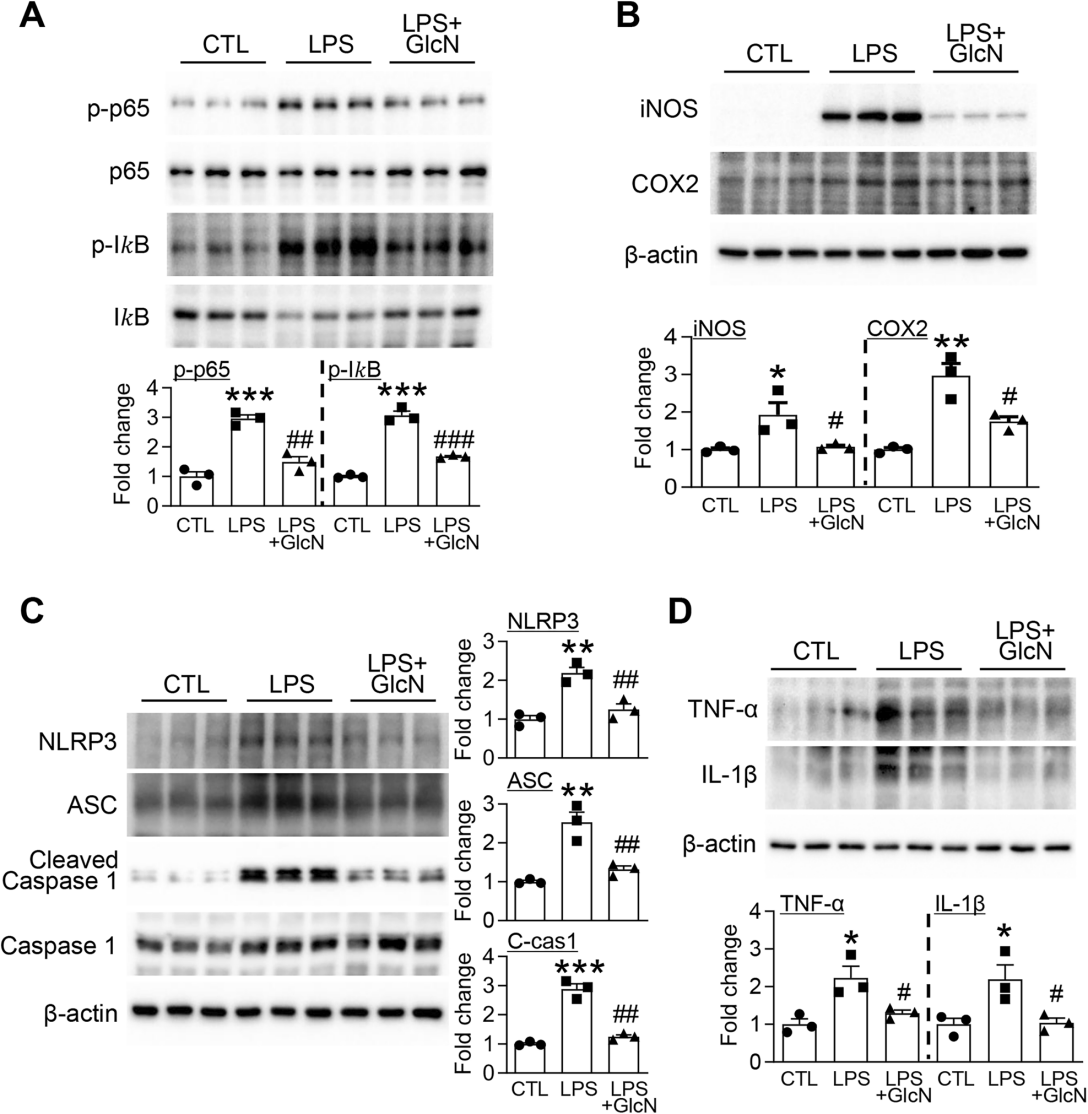
Restoration of *O*-GlcNAcylation in microglia attenuates LPS-Induced inflammatory and inflammasome signaling. BV2 cells were stimulated with LPS (400 ng/mL) for 24 h in the presence or absence of GlcN (5 mM). **A** Western blot analysis of NF-κB signaling components, including phosphorylated and total p65 and IκB. Phosphorylation levels were normalized to total protein (*n* = 3 per group). **B** Western blot analysis of pro-inflammatory enzymes iNOS and COX-2, normalized to β-actin (*n* = 3 per group). **C** Western blot assessment of inflammasome activation by measuring NLRP3, ASC, and cleaved caspase-1, with cleaved caspase-1 normalized to total caspase-1 and NLRP3/ASC normalized to β-actin (*n* = 3 per group). **D** Western blot analysis of inflammatory cytokines TNF-α and IL-1β, normalized to β-actin (*n* = 3 per group). Data are presented as mean SEM; *p < 0.05, **p < 0.01, ***p < 0.001 versus control, ^#^*p* < 0.05, ^##^*p* < 0.01, ^###^*p* < 0.001 versus LPS. Statistical analysis was performed using one-way ANOVA with Tukey’s post hoc multiple comparison test.

Building on our in vivo observations that LPS-induced hippocampal neuroinflammation is associated with a pronounced reduction in microglial *O*-GlcNAcylation, we next investigated whether direct restoration of *O-*GlcNAc levels in microglia could modulate inflammatory signaling and inflammasome activation using microglial BV2 cells. LPS exposure elicited robust activation of canonical NF-κB signaling, as indicated by increased phosphorylation of p65 and IκB (Fig. 5A). Concurrently, expression of pro-inflammatory effectors, including iNOS and COX-2, was markedly upregulated. LPS also triggered substantial inflammasome activation, evidenced by elevated levels of NLRP3 and cleaved caspase-1 (Fig. 5B, C), and promoted a strong pro-inflammatory cytokine response, as demonstrated by increased TNF-α and IL-1β secretion (Fig. 5D). Importantly, co-treatment with GlcN effectively restored *O*-GlcNAcylation in BV2 cells (Fig. 4B), which was accompanied by a robust attenuation of LPS-induced NF-κB activation, suppression of iNOS and COX-2 expression, and downregulation of inflammasome components. Furthermore, GlcN treatment normalized TNF-α and IL-1β levels, highlighting its ability to reestablish cytokine homeostasis under inflammatory conditions (Fig. 5A–D). Collectively, these data suggest that microglial *O*-GlcNAcylation may represent an important regulatory node associated with the modulation of canonical inflammatory signaling and inflammasome-related responses.

### GlcN restores O-GlcNAcylation of NF-κB subunits and suppresses their nuclear translocation in microglia

**Fig. 6:**
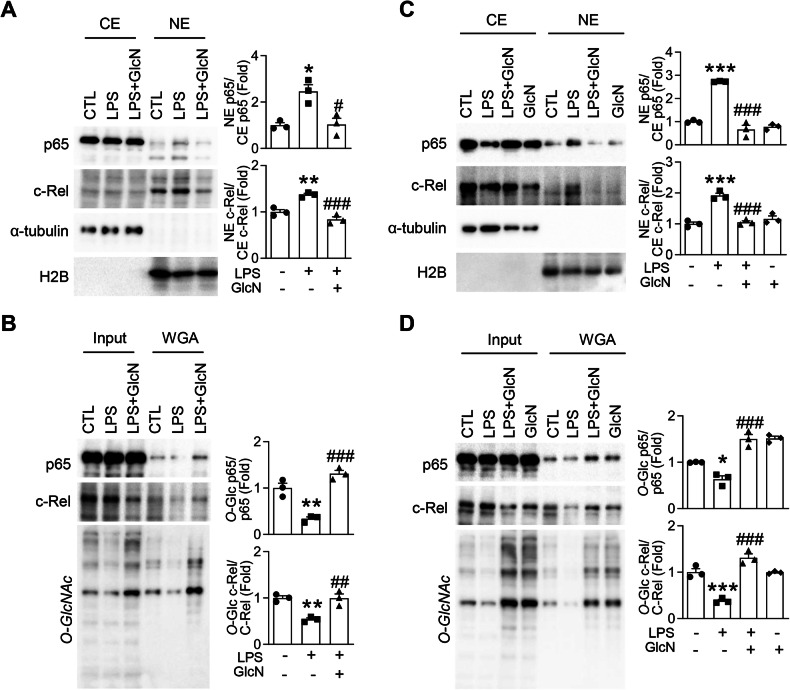
GlcN restores *O*-GlcNAcylation of NF-κB subunits and suppresses their nuclear translocation in microglia. **A**, **B** BV2 microglial cells were treated with LPS (400 ng/mL) for 24 h in the presence or absence of GlcN (5 mM). **A** Nuclear (NE) and cytoplasmic (CE) fractions were isolated to examine the subcellular distribution of NF-κB subunits p65 and c-Rel by western blot. Quantification of nuclear translocation was normalized to the corresponding cytoplasmic levels (*n* = 3 per group). **B** Wheat germ agglutinin (WGA) pull-down assays were performed on total cell lysates to evaluate *O*-GlcNAcylation of p65 and c-Rel. *O-GlcNAc*-modified proteins were detected by western blot and normalized to total p65 or c-Rel (*n* = 3 per group). **C**, **D** In vivo experiments: LPS (15 μg) was stereotaxically injected into the lateral ventricle of mouse brains. GlcN (200 mg/kg) was administered intraperitoneally three times per week for 4 weeks. **C** Cytoplasmic (CE) and nuclear (NE) protein fractions were isolated from hippocampal tissue. Western blot analysis was performed for NF-κB subunits p65 and c-Rel. Nuclear levels were normalized to corresponding cytoplasmic fractions. Histone H2B and α-tubulin served as nuclear and cytoplasmic markers, respectively (*n* = 3 per group). **D** WGA pull-down assays were performed on total hippocampal lysates to assess *O*-GlcNAcylation of p65 and c-Rel. *O-GlcNAc*-modified proteins were detected by western blot and normalized to total protein levels (*n* = 3 per group). Data are presented as mean SEM; *p < 0.05, **p < 0.01, ***p < 0.001 versus control, ^#^*p* < 0.05, ^##^*p* < 0.01, ^###^*p* < 0.001 versus LPS. Statistical analysis was performed using one-way ANOVA with Tukey’s post hoc multiple comparison test.

To delineate the molecular mechanisms by which *O*-GlcNAcylation regulates microglial inflammatory signaling, we examined the nuclear translocation of canonical NF-κB subunits, p65 and c-Rel, in BV2 microglial cultures. LPS stimulation induced robust nuclear accumulation of both transcription factors, consistent with activation of NF-κB signaling. Remarkably, GlcN treatment significantly attenuated the nuclear translocation of p65 and c-Rel, indicating that enhancement of *O*-GlcNAcylation can constrain NF-κB pathway activation at the level of subcellular localization (Fig. 6A). To investigate whether these effects involve direct modification of NF-κB subunits, we performed WGA lectin pull-down assays. LPS exposure markedly reduced the *O*-GlcNAcylation of p65 and c-Rel, whereas GlcN co-treatment restored their *O*-GlcNAcylation levels to baseline (Fig. 6B). These findings suggest that *O*-GlcNAcylation serves as a post-translational modification that modulates NF-κB transcriptional activity by regulating nuclear translocation. To corroborate these findings in vivo, we analyzed hippocampal cytoplasmic and nuclear fractions from an LPS-induced neuroinflammation model relevant to AD. In agreement with our in vitro observations, LPS challenge markedly increased the nuclear accumulation of p65 and c-Rel, an effect that was substantially attenuated by systemic GlcN administration (Fig. 6C). Furthermore, the LPS-induced decrease in *O*-GlcNAcylation of these NF-κB subunits was completely restored by GlcN treatment in brain tissue (Fig. 6D). Collectively, these findings indicate that NF-κB subunits p65 and c-Rel are *O*-GlcNAcylated in microglia, and that changes in this modification under inflammatory conditions are associated with altered nuclear translocation and enhanced pro-inflammatory signaling.

### GlcN-mediated enhancement of O-GlcNAcylation reprograms LPS-induced microglial polarization toward an anti-inflammatory phenotype

**Fig. 7:**
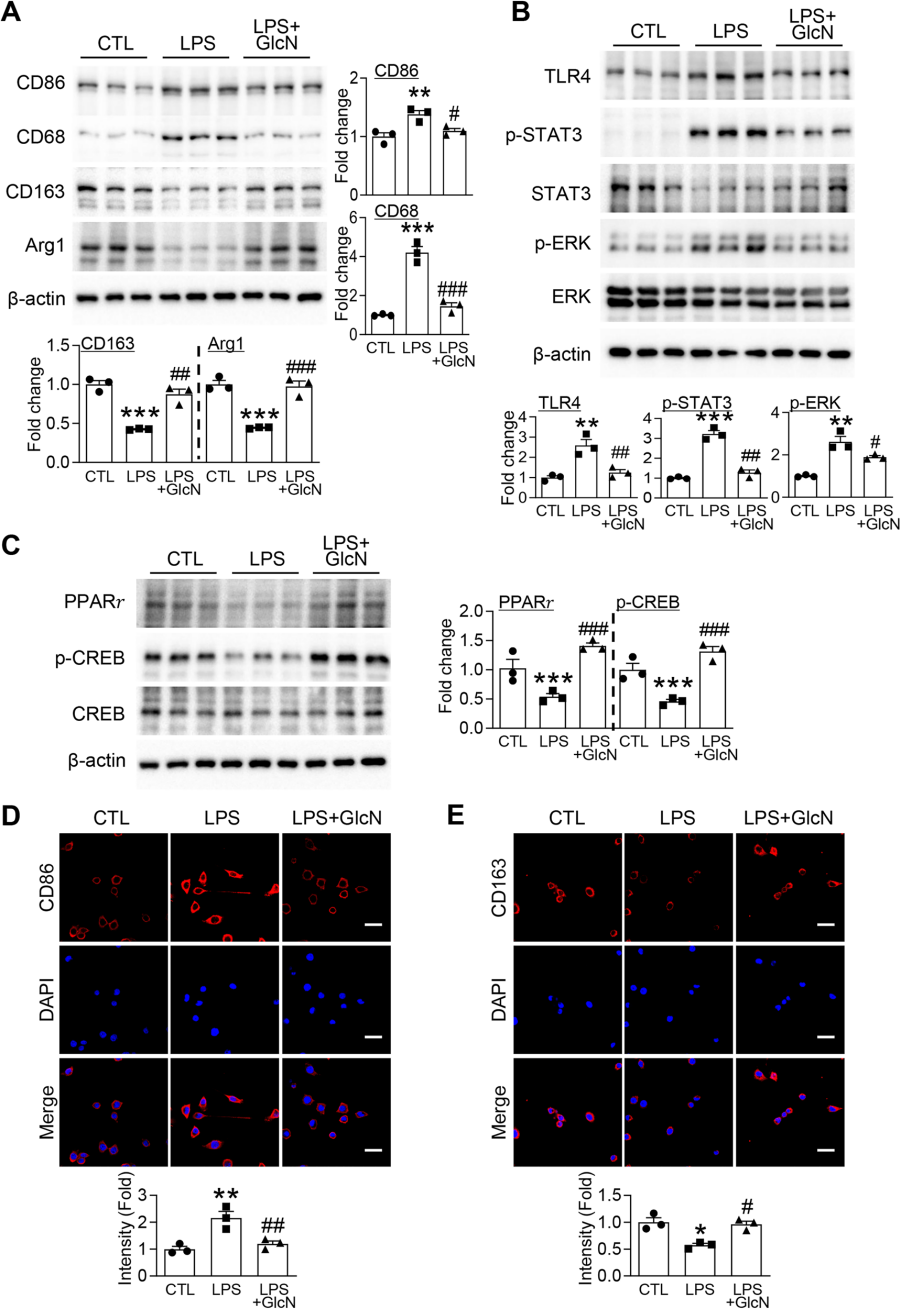
GlcN-mediated enhancement of *O*-GlcNAcylation reprograms LPS-induced microglial polarization toward an anti-inflammatory phenotype. BV2 microglial cells were stimulated with LPS (400 ng/mL) for 24 h in the presence or absence of GlcN (5 mM). **A** Western blot analysis of M1 (CD86, CD68) and M2 (CD163, Arg1) microglial markers. β-actin was used as a loading control, and protein expression levels were quantified and normalized to β-actin (*n* = 3 per group). **B** Western blot assessment of TLR4/STAT3/ERK signaling. Phosphorylation levels of STAT3 and ERK were determined using phospho-specific antibodies, with ratios of phosphorylated to total protein (p-STAT3/STAT3, p-ERK/ERK) calculated and normalized. TLR4 expression was quantified and normalized to β-actin (*n* = 3 per group). **C** Western blot analysis of M2-associated transcriptional regulators PPARγ and phosphorylated CREB (p-CREB). p-CREB levels were normalized to total CREB, and PPARγ levels were normalized to β-actin (*n* = 3 per group). Representative immunofluorescence images of microglial cells. CD86 (red) and DAPI (blue) are shown in (**D**), and CD163 (red) with DAPI (blue) in (**E**). Scale bars = 10 μm (*n* = 3 per group). Data are presented as mean SEM; ^**^p < 0.01, ^***^p < 0.001 versus control, ^#^*p* < 0.05, ^##^*p* < 0.01, ^###^*p* < 0.001 versus LPS. Statistical analysis was performed using one-way ANOVA with Tukey’s post hoc multiple comparison test.

Microglial functional polarization, a dynamic process governed by intracellular signaling cascades such as NF-κB, is broadly classified into a pro-inflammatory M1 state and an anti-inflammatory M2 state, each characterized by distinct transcriptional and protein expression profiles. In BV2 microglial cells, LPS stimulation elicited a pronounced shift toward the M1 phenotype, as reflected by robust upregulation of canonical M1 markers (CD86, CD68) and a concomitant suppression of M2-associated proteins (Arg1, CD163) (Fig. 7A). This pro-inflammatory polarization was accompanied by activation of M1-linked signaling pathways, including increased TLR4 expression and phosphorylation of STAT3 and ERK (Fig. 7B), alongside downregulation of M2-associated transcriptional regulators, notably PPARγ and phosphorylated CREB (p-CREB) (Fig. 7C). Elevation of *O*-GlcNAcylation *via* GlcN treatment markedly counteracted these LPS-induced changes, reducing M1 marker expression while restoring M2-associated proteins to near-basal levels (Fig. 7A). At the signaling level, GlcN attenuated phosphorylation of STAT3 and ERK, while reinstating PPARγ and p-CREB expression (Fig. 7B, C), indicative of a transcriptional environment favoring M2 polarization. Immunofluorescence analyses corroborated these biochemical findings, showing that GlcN prevented LPS-induced CD86 upregulation and restored CD163 expression (Fig. 7D, E). Taken together, these findings identify *O*-GlcNAcylation as a previously unrecognized regulatory axis in microglial phenotypic plasticity.

### GlcN reprograms microglial polarization and signaling networks in the hippocampus in an LPS-induced neuroinflammation model relevant to AD

**Fig. 8:**
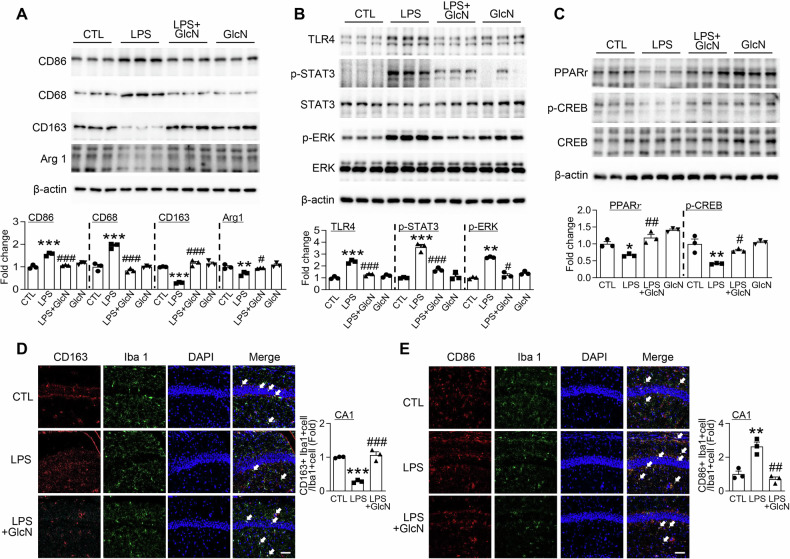
GlcN reprograms microglial polarization and signaling networks in the hippocampus of LPS-induced AD-relevant neuroinflammatory mice. LPS (15 μg) was stereotaxically injected into the lateral ventricle of the mouse brains. GlcN (200 mg/kg) was administered intraperitoneally three times per week for four weeks. **A** Western blot analysis of hippocampus lysates was performed to evaluate microglial polarization markers. M1 markers (CD86, CD68) and M2 markers (CD163, Arg1) were quantified, and expression levels were normalized to β-actin (*n* = 3 per group). **B** Western blot analysis of TLR4/STAT3/ERK signaling. Phosphorylation of STAT3 and ERK was assessed using phospho-specific antibodies, and ratios of phosphorylated to total protein (p-STAT3/STAT3, p-ERK/ERK) were calculated. TLR4 expression was quantified and normalized to β-actin (*n* = 3 per group). **C** Western blot analysis of M2-associated transcription factors PPARγ and phosphorylated CREB (p-CREB). p-CREB levels were normalized to total CREB, and PPARγ levels were normalized to β-actin (*n* = 3 per group). **D**, **E** Representative immunofluorescence images of hippocampal microglia. CD163 (red) with Iba1 (green) and DAPI (blue) are shown in (**D**) to visualize M2 microglial phenotypes (arrowhead). CD86 (red) with Iba1 (green) and DAPI (blue) are shown in (**E**) to visualize M1 microglial phenotypes (arrowhead). Scale bars = 50 μm (*n* = 3 per group). Data are presented as mean SEM; *p < 0.05, **p < 0.01, ***p < 0.001 versus control, ^#^*p* < 0.05, ^##^*p* < 0.01, ^###^*p* < 0.001 versus LPS. Statistical analysis was performed using one-way ANOVA with Tukey’s post hoc multiple comparison test.

We examined the hippocampal expression of phenotype-specific markers and their upstream signaling mediators in an LPS-Induced neuroinflammation model relevant to AD. LPS administration elicited a robust pro-inflammatory reprogramming of hippocampal microglia, evidenced by increased expression of canonical M1 markers (CD86, CD68) and a concomitant downregulation of M2-associated proteins (Arg1, CD163) (Fig. 8A). This phenotypic skewing was paralleled by activation of M1-linked signaling pathways, as indicated by elevated TLR4 expression and hyperphosphorylation of STAT3 and ERK, alongside suppression of M2-promoting regulators, including PPARγ and phosphorylated CREB (Fig. 8B, C). Notably, pharmacological enhancement of *O*-GlcNAcylation through systemic GlcN administration markedly attenuated LPS-induced M1 polarization, restoring the expression of M2 markers to near-baseline levels (Fig. 8A) and rebalancing the associated signaling network by downregulating TLR4 and p-STAT3/p-ERK while concomitantly elevating PPARγ and p-CREB (Fig. 8B, C). Immunofluorescence analyses corroborated these biochemical findings, revealing that GlcN reversed the LPS-induced increase in M1 marker immunoreactivity and restored M2 marker expression within hippocampal microglia (Fig. 8D, E and Fig. S3A–D). These results provide the first in vivo evidence that *O*-GlcNAcylation serves as a pivotal molecular switch governing microglial polarization and its upstream signaling architecture in the inflamed AD brain.

## Discussion

In the present study, we show that dysregulation of protein *O*-GlcNAcylation is associated with neuroinflammatory responses and microglial dysfunction in AD, and that enhancement of this post-translational modification by GlcN is linked to attenuated inflammatory signaling and neuroprotective effects in both in vitro and in vivo neuroinflammation models relevant to AD. Postmortem analysis of AD hippocampal tissue revealed a marked reduction in global *O*-GlcNAcylation, particularly within Iba1-positive microglia, correlating with enhanced NF-κB activity, NLRP3 inflammasome activation, and upregulation of classical M1 markers. These observations provide strong evidence for a link between impaired *O-*GlcNAc homeostasis and a pro-inflammatory microglial phenotype in human AD. However, given the limited number of postmortem cases analyzed and the sensitivity of *O*-GlcNAcylation to variables such as postmortem interval, Braak and CERAD stage, and medical comorbidities, these findings should be interpreted as exploratory and warrant validation in larger, well-characterized cohorts.

Our in vivo and in vitro experiments further establish the mechanistic relevance of *O*-GlcNAcylation in regulating neuroinflammatory responses. Building upon our previous studies demonstrating that inflammation from diverse etiologies impairs cerebral *O*-GlcNAcylation [25–28], the present study provides more direct evidence to substantiate this relationship. Specifically, we demonstrate that direct hippocampal administration of LPS markedly decreases *O*-GlcNAcylation within the hippocampal tissue and, more importantly, within resident microglia themselves. These findings suggest that neuroinflammation is associated with a reduction in *O*-GlcNAc cycling in microglia, rather than reflecting only a secondary consequence of systemic immune activation. Furthermore, GlcN treatment restored *O*-GlcNAcylation and was associated with a reversal of pro-inflammatory polarization, suggesting a potential link between *O*-GlcNAc dynamics and microglial activation states. Mechanistically, we show that *O*-GlcNAcylation modulates NF-κB signaling, as LPS promoted nuclear translocation of p65 and c-Rel, whereas restoration of *O-*GlcNAc by GlcN attenuated their nuclear accumulation and reduced downstream transcription of pro-inflammatory mediators, including iNOS, COX-2, TNF-α, and IL-1β. These mechanistic findings also extend prior work that primarily focused on global *O*-GlcNAc elevation and necroptosis-related pathways [23]. In contrast, our results identify a distinct *O*-GlcNAc–dependent regulatory mechanism involving NF-κB and NLRP3 inflammasome signaling, as well as microglia-specific reductions in *O*-GlcNAcylation observed in human AD tissue. This distinction highlights the novelty of our study and underscores the broader role of *O*-GlcNAcylation in shaping microglial activation beyond the necroptosis-centered framework described previously. Although our findings demonstrate that reduced *O*-GlcNAcylation is associated with enhanced microglial inflammatory signaling, definitive causality cannot be concluded. Future studies employing microglia-specific genetic modulation of OGT or OGA will be required to determine whether changes in *O*-GlcNAcylation directly drive microglial activation. Our data also showed increased GFAP expression, indicating reactive astrocytosis in the hippocampus. Activated astrocytes can modulate neuroinflammation, synaptic function, and metabolism, potentially amplifying microglial responses [29–31]. Thus, concurrent astroglial activation may contribute to the overall neuroinflammatory environment and interact with *O*-GlcNAcylation–dependent regulation of glial function.

Beyond the regulation of NF-κB signaling, our findings indicate that *O*-GlcNAcylation exerts a broader influence on microglial phenotypic plasticity. Reflecting its role as a master metabolic sensor, *O*-GlcNAcylation may contribute to the regulation of microglial polarization, potentially influencing the balance between pro-inflammatory and anti-inflammatory states. LPS exposure drove microglia toward an M1-like phenotype through the activation of TLR4/STAT3/ERK pathways and concomitant suppression of M2-associated transcriptional regulators, including PPARγ and phosphorylated CREB. Restoration of *O*-GlcNAcylation by GlcN effectively reversed these alterations, promoting M2-associated pathways and fostering a more anti-inflammatory transcriptional landscape. While we have described microglial activation using the conventional M1/M2 framework for clarity, we acknowledge that this binary classification oversimplifies the spectrum of microglial states observed in vivo. Recent single-cell transcriptomic studies have demonstrated that microglia adopt diverse, disease-associated phenotypes that extend beyond the classical M1/M2 distinction, including disease-associated, interferon-responsive, and lipid-processing subsets [32–34]. It is therefore likely that *O*-GlcNAcylation regulates a continuum of activation states rather than discrete phenotypic categories. Further investigations employing single-cell or spatial transcriptomic approaches will be necessary to define how *O*-GlcNAc signaling modulates these heterogeneous microglial populations in the context of neurodegeneration.

Consistent with the observed molecular and cellular improvements, these changes were accompanied by corresponding benefits at the behavioral level. Immunomodulatory reprogramming translated into preserved neuronal integrity, maintenance of dendritic architecture, and stabilization of synaptic marker expression within the hippocampus, ultimately supporting improved performance in hippocampus-dependent cognitive tasks. Although escape latency during Morris water maze acquisition did not differ among groups, this parameter is relatively insensitive to hippocampal dysfunction, as mice can locate the hidden platform through nondirected exploratory swimming during early training. In contrast, memory-dependent tasks that assess consolidation and retrieval—including passive avoidance, novel object recognition, and the probe trial—are more sensitive to LPS-induced hippocampal inflammation and more effectively reveal the neuroprotective effects of restored *O*-GlcNAcylation.

Although these neuroprotective outcomes likely arise, at least in part, as secondary effects of attenuated neuroinflammation, it remains possible that *O*-GlcNAcylation exerts direct protective actions on neuronal and synaptic components independent of its anti-inflammatory functions. For example, previous studies have reported that *O*-GlcNAcylation modifies tau and synaptic proteins [35, 36], suggesting that the dual anti-inflammatory and neuroprotective effects observed here may reflect both cell-autonomous neuronal mechanisms and microglia-mediated regulation. Future studies employing cell-type-specific manipulation of *O-*GlcNAc cycling will be required to delineate the relative contribution of these parallel mechanisms.

While these findings underscore the therapeutic potential of targeting *O*-GlcNAcylation, several limitations warrant consideration. Although GlcN treatment effectively restored *O*-GlcNAcylation and attenuated neuroinflammation, several pharmacological limitations should be acknowledged. GlcN is not a specific modulator of *O*-GlcNAc cycling and exhibits relatively limited penetration across the blood–brain barrier, raising uncertainty as to whether its effects observed here result from direct modulation of microglial *O*-GlcNAcylation or from indirect systemic or metabolic influences. Therefore, while our findings support a potential neuroprotective role of *O*-GlcNAc enhancement, these results should be interpreted cautiously, and claims of direct therapeutic efficacy remain preliminary. Future studies incorporating pharmacokinetic validation and the use of brain-permeable, selective OGA inhibitors or genetic tools will be required to confirm the specificity and central mechanism of action. Although GlcN is a widely used enhancer of *O*-GlcNAcylation, its actions are not entirely specific and may influence glucose metabolism or diabetes-related pathways [37, 38]. Nonetheless, its favorable safety profile and function as an endogenous intermediate of the HBP suggest that GlcN may serve as a physiologically relevant and biologically meaningful alternative to synthetic OGA inhibitors. To delineate the specificity of these effects, future studies incorporating selective pharmacological OGA modulators, as well as genetic strategies such as microglia-specific knockdown or knockout of OGA in vivo, will be essential to establish the direct causal role of *O*-GlcNAcylation in regulating neuroinflammation and microglial polarization. Moreover, the LPS-induced neuroinflammation paradigm used here captures important aspects of AD-related microglial activation but does not fully recapitulate the complex pathophysiology of human AD, underscoring the need for validation in transgenic amyloid- and tau-based models. Additional contributions from astrocytes, neurons, and peripheral immune cells also cannot be excluded, emphasizing the importance of dissecting cell-type-specific interactions in future work. Finally, although restoration of *O*-GlcNAcylation was associated with microglial M2 polarization, neuronal preservation, and improved cognitive function, the mechanistic links between molecular, cellular, and behavioral outcomes remain to be fully defined. Future studies integrating cell-type-specific manipulations with longitudinal in vivo analyses will be necessary to establish causal relationships and clarify the temporal dynamics of *O*-GlcNAc–mediated neuroprotection.

In conclusion, our findings suggest that reduced *O*-GlcNAcylation is associated with enhanced microglial pro-inflammatory activation and neuronal impairment in an acute neuroinflammation model relevant to AD-associated inflammation. Enhancement of *O*-GlcNAcylation appeared to attenuate NF-κB and inflammasome signaling, favoring an anti-inflammatory microglial phenotype, supporting neuronal preservation and improved cognitive outcomes. Collectively, these findings expand upon previous work by identifying microglia-specific *O*-GlcNAc dysregulation and a distinct NF-κB/NLRP3–centered mechanism, thereby emphasizing the novelty and therapeutic relevance of *O*-GlcNAcylation in regulating neuroinflammatory processes.

## Supplementary information

Supplementary Data

Western bolt original data

## Data Availability

The data and materials used in this research are available upon request from the corresponding author.

## Code availability

No custom computer code was used in this study.
