# Supplementary material for: O-GlcNAcylation reprograms microglial inflammatory states and attenuates Alzheimer’s disease pathology
Source: Cell Death Dis. 2026 May 21;17(1):638. doi: 10.1038/s41419-026-08862-3 (PMC13365447; doi:10.1038/s41419-026-08862-3)
Supplement: Supplementary file 1 — Supplementary Data [file 41419_2026_8862_MOESM1_ESM.pdf]

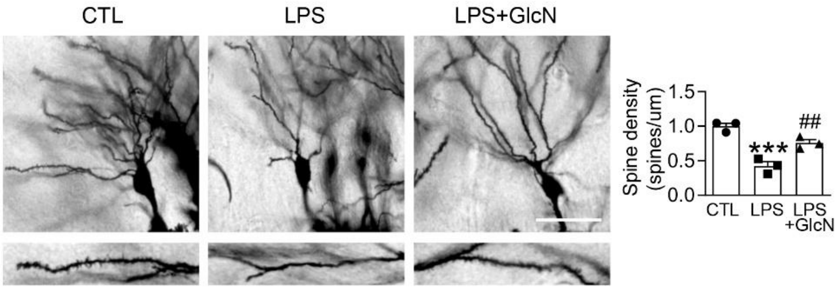

Supplementary Figure 1. Representative Golgi–Cox–stained images of basal dendrites from hippocampal pyramidal neurons

Representative images of dendritic morphology in hippocampal CA1 visualized by Golgi staining. Scale bar = 10 μm (n = 3 per group). Data are presented as mean SEM; $^{***}p<0.001$ versus control, $^{\#\#}p<0.01$ versus LPS. Statistical analysis was performed using one-way ANOVA with Tukey's post hoc multiple comparison test.

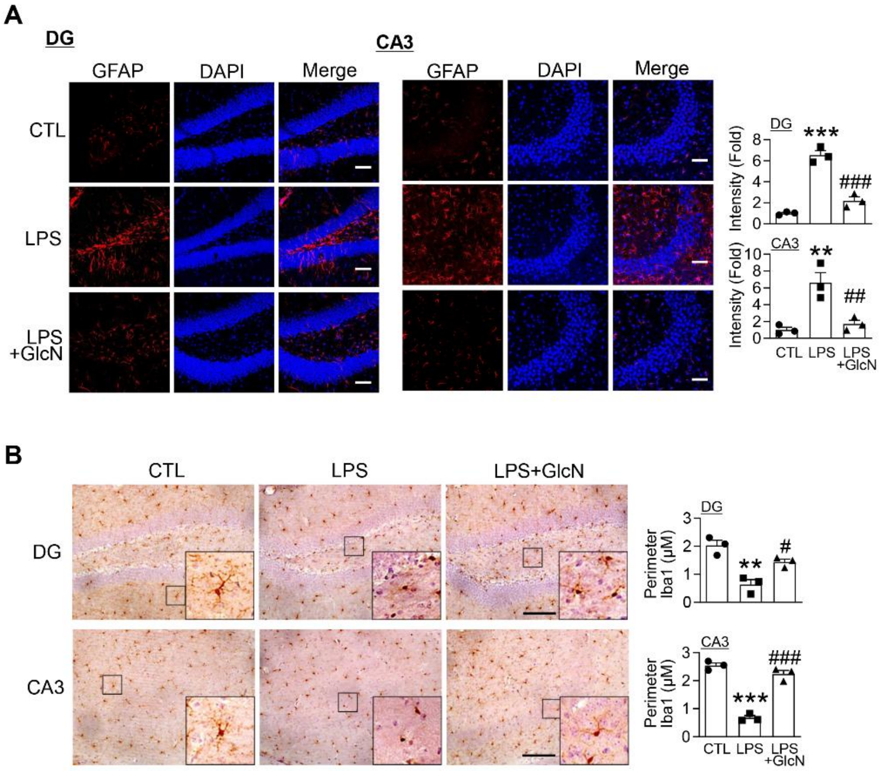

Supplementary Figure 2. GlcN suppresses glial activation induced by LPS

Mice received ICV injections of LPS (15 μg) into the lateral ventricle, and GlcN was

administered intraperitoneally at 200 mg/kg, three times per week for 4 weeks. (A) Representative immunofluorescence images of the hippocampus showing GFAP (red) and DAPI (blue). Scale bar = 50 μm (n = 3 per group). (B) Iba1 immunohistochemistry to assess microglial morphology in the hippocampus. Scale bar = 100 μm (n = 3 per group). Data are presented as mean SEM; $^{**}p<0.01$, $^{***}p<0.001$ versus control, $^{\#}p<0.05$, $^{\#\#}p<0.01$, $^{\#\#\#}p<0.001$ versus LPS. Statistical analysis was performed using one-way ANOVA with Tukey's post hoc multiple comparison test.

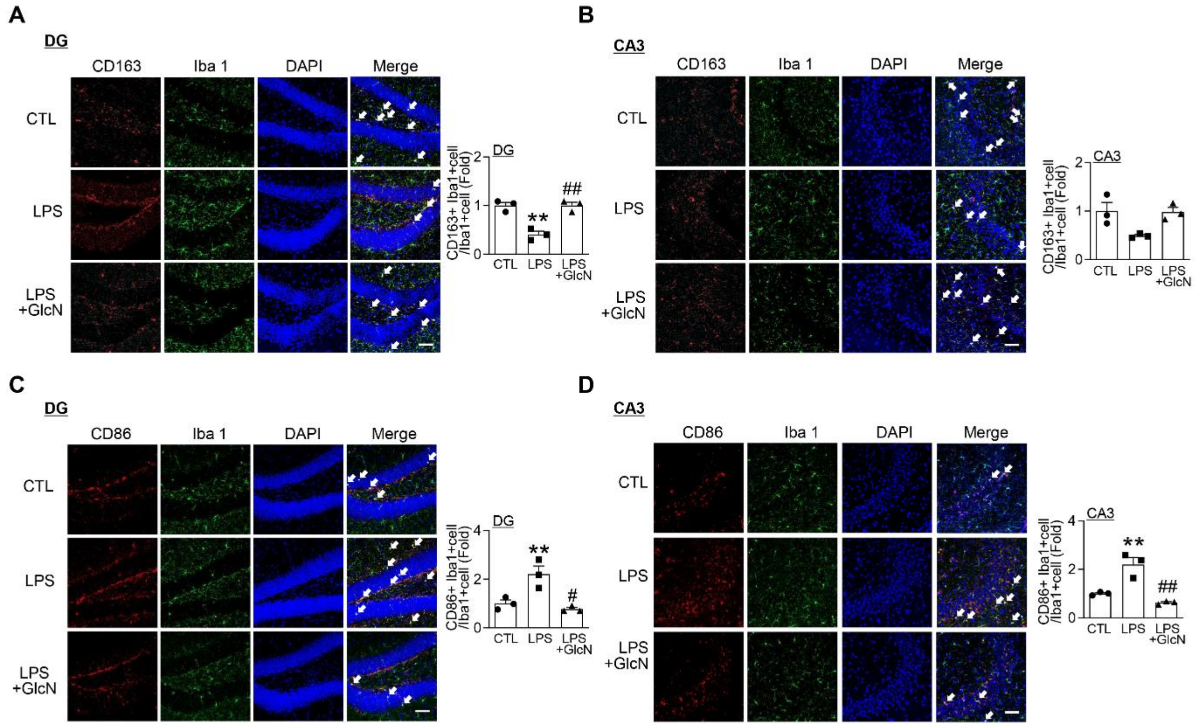

Supplementary Figure 3. GlcN reprograms microglial polarization in the hippocampus of LPS-Induced AD-relevant neuroinflammatory mice

LPS (15 μg) was stereotaxically injected into the lateral ventricle of mouse brains. GlcN (200 mg/kg) was administered intraperitoneally three times per week for four weeks. (A–B) Representative immunofluorescence images of hippocampal microglia. CD163 (red) with Iba1 (green) and DAPI (blue) are shown in panel A to visualize M2 microglial phenotypes

(arrowhead). CD86 (red) with Iba1 (green) and DAPI (blue) are shown in panel B to visualize M1 microglial phenotypes (arrowhead). Scale bars = 50 μm (n = 3 per group). Data are presented as mean SEM; $^{**}p<0.01$, $^{***}p<0.001$ versus control, $^{##}p<0.01$, $^{###}p<0.001$ versus LPS. Statistical analysis was performed using one-way ANOVA with Tukey's post hoc multiple comparison test.

NO.	Gender	Age	Postmortem interval (h)	Clinical diagnosis name	Autopsy diagnosis name	Thal phase for amyloid plaques by IHC (A score)	Braak stage for neurofibrillary degerneration (B score)	NIA-AA Alzheimer's disease neuropathologic change (ADNC)	hippocampal subregion
1	Male	50	28h	depression	Near Normal brain	Phase 0 (A0)	Stage 0 (B0)	Not AD	CA1,2,3,4+dentate gyrus
2	Male	72	2h	Normal brain	Normal brain	Phase 0 (A0)	Stage 0 (B0)	Not AD	CA1,2,3,4+dentate gyrus
3	Female	63	13h	Ovarian carcinoma	Normal brain	Phase 0 (A0)	Stage 0 (B0)	Not AD	CA1,2,3,4+dentate gyrus
4	Female	78	6.2h	Acute heart failure	Alzheimer disease	Phase 3 (A2)	Stage V (B3)	Intermediate ADNC	CA1,2,3,4+dentate gyrus
5	Female	76	4h	Parkinsonism	AD + Synucleinopathy	Phase 4 (A3)	Stage VI (B3)	High ADNC	CA1,2,3,4+dentate gyrus
6	Male	61	2h 30m	MSA	Dementia with Lewy bodies	Phase 5 (A3)	Stage VI (B3)	High ADNC	CA1,2,3,4+dentate gyrus

Supplementary Table 1. Clinical and neuropathological characteristics of human brain donors used in this study

This table summarizes demographic information and neuropathological features of all human cases included in the analysis. Variables presented include age at death, sex, Braak neurofibrillary tangle stage, amyloid plaque score, and total tangle burden as assessed by neuropathological examination. AD cases exhibited advanced Braak stages and high amyloid/tangle pathology, while control cases showed no significant Alzheimer's disease–related pathology. These data provide detailed characterization of the limited human samples (n = 3 per group) used in Figures 1 and related analyses.
