# Supplementary figures and images for: O-GlcNAcylation reprograms microglial inflammatory states and attenuates Alzheimer’s disease pathology
Source: Cell Death Dis. 2026 May 21;17(1):638. doi: 10.1038/s41419-026-08862-3 (PMC13365447; doi:10.1038/s41419-026-08862-3)

Figure 1

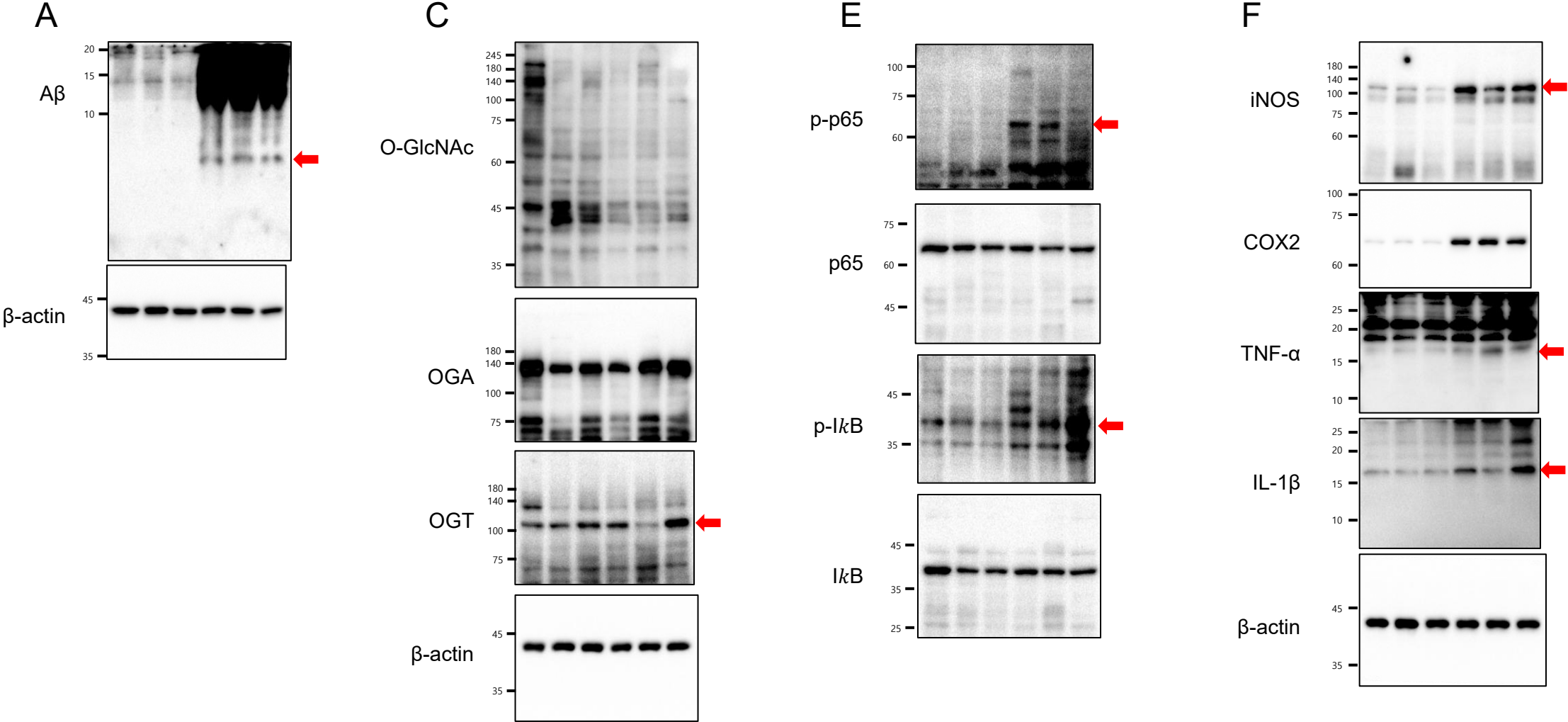

Figure 1

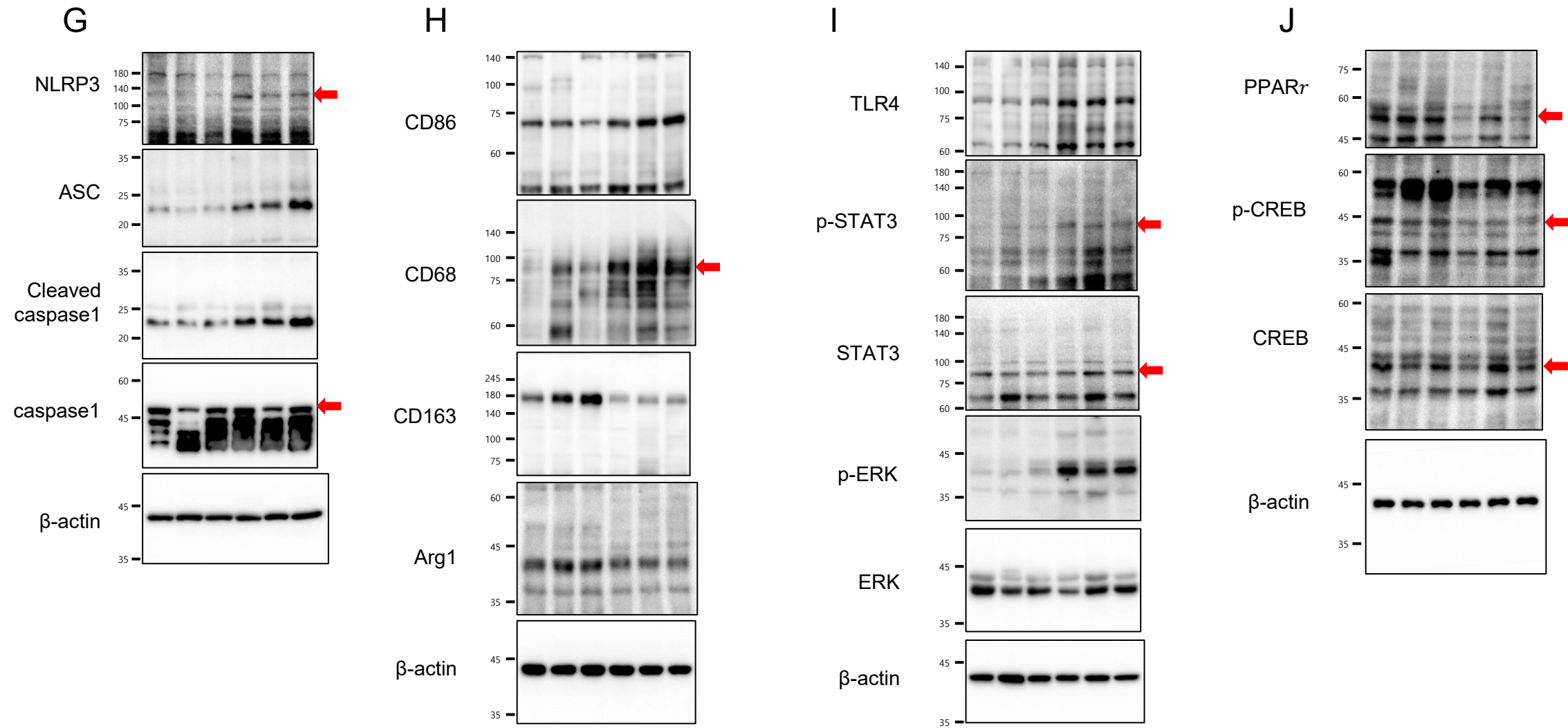

Figure 2

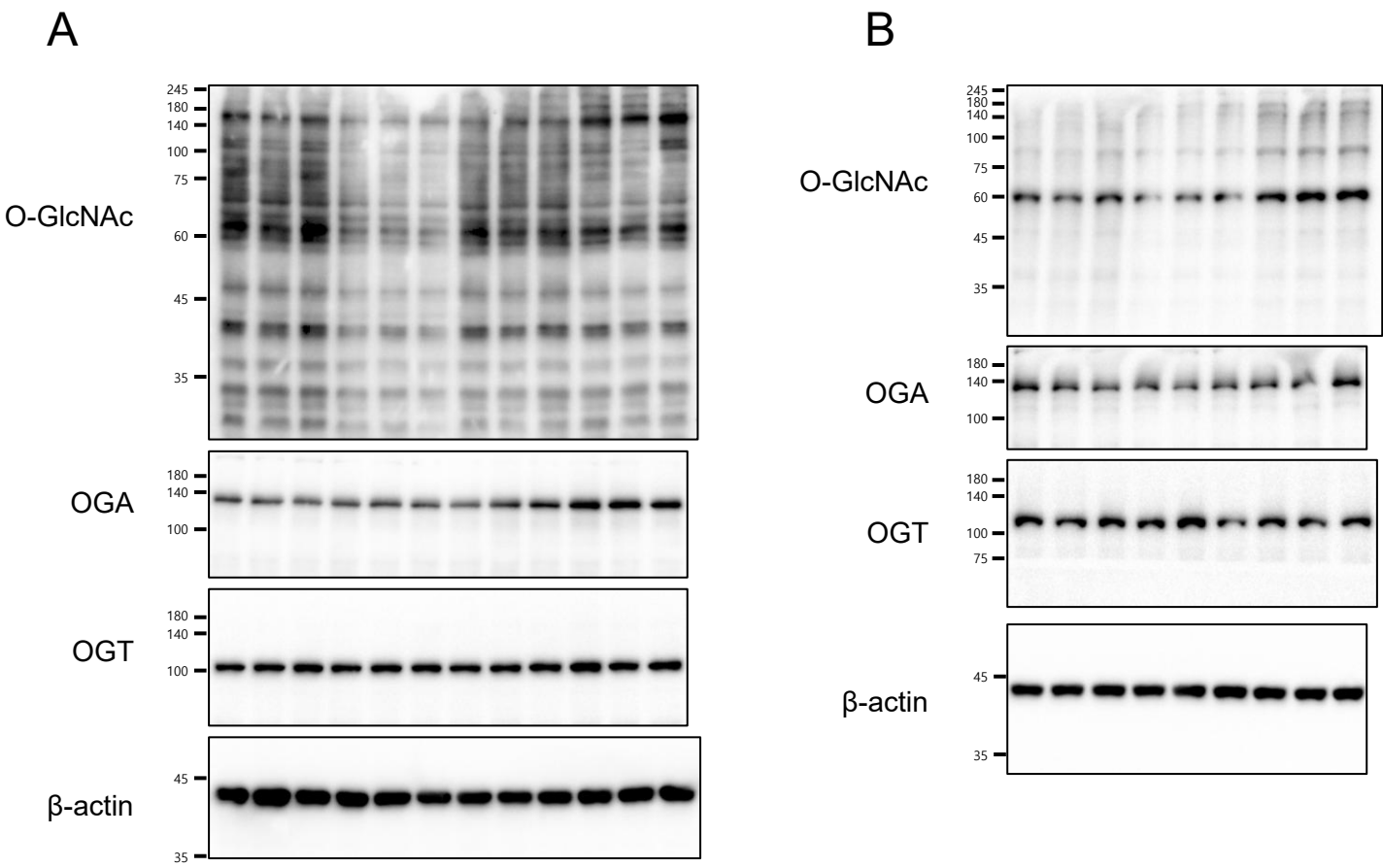

Figure 4

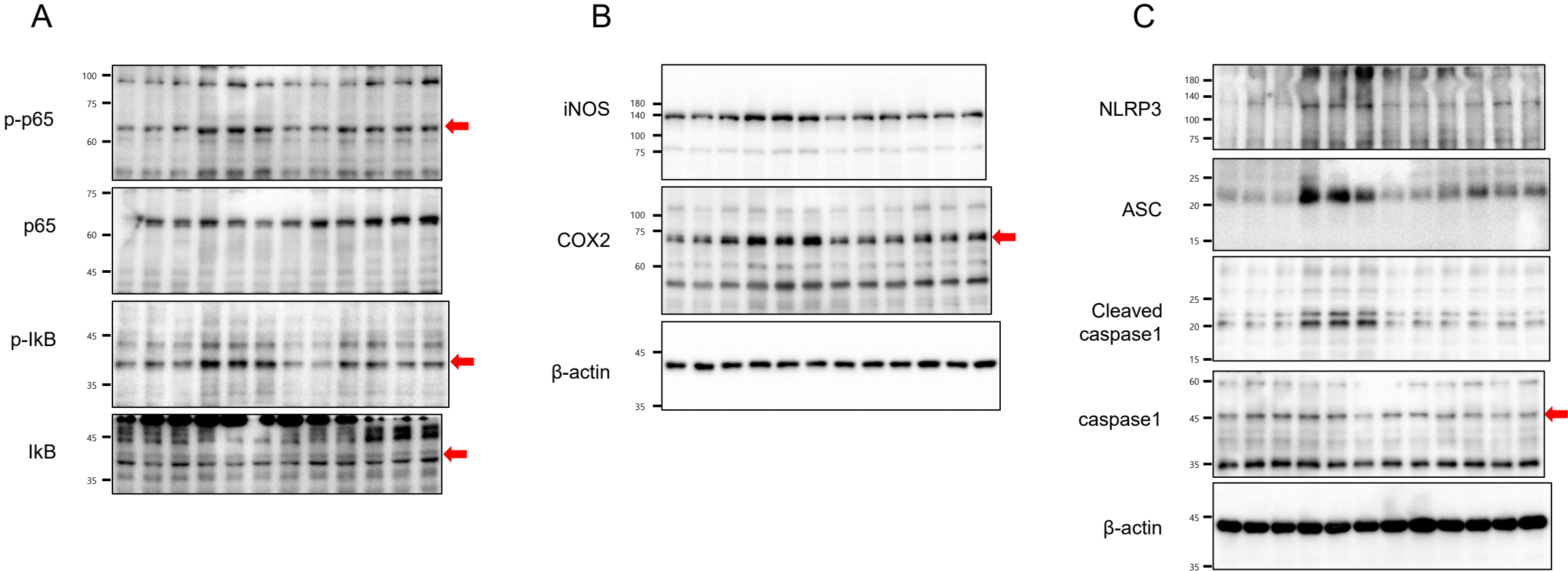

Figure 4

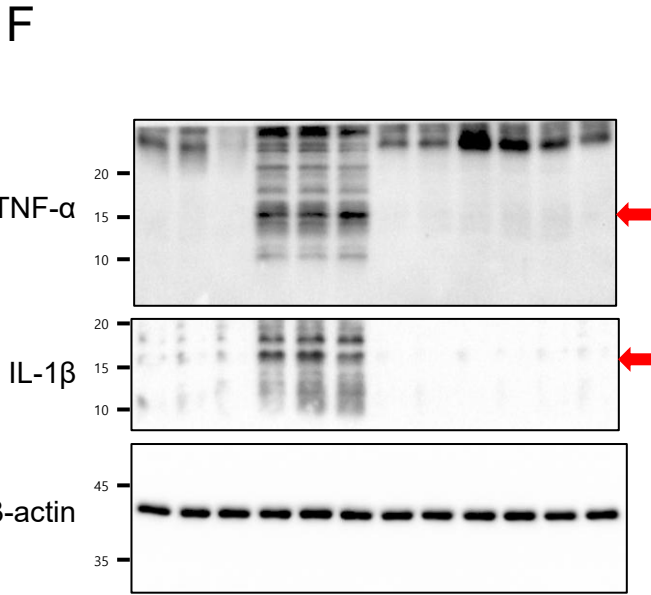

Figure 5

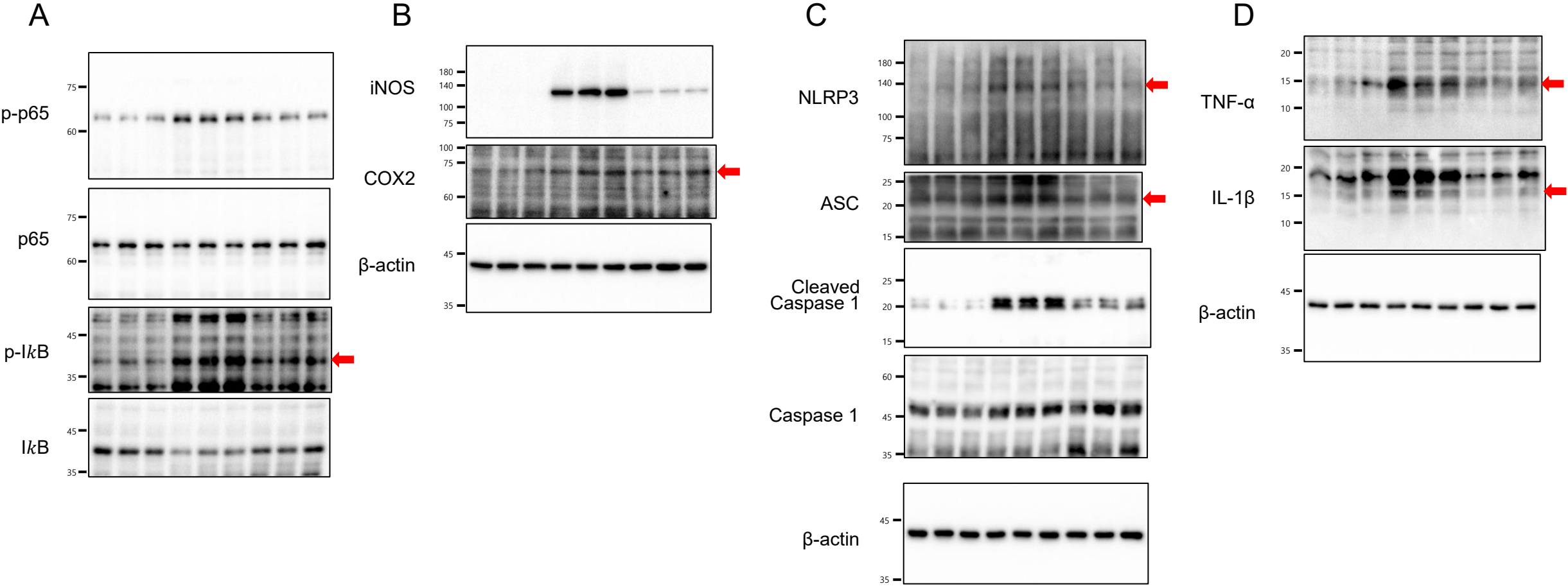

Figure 6

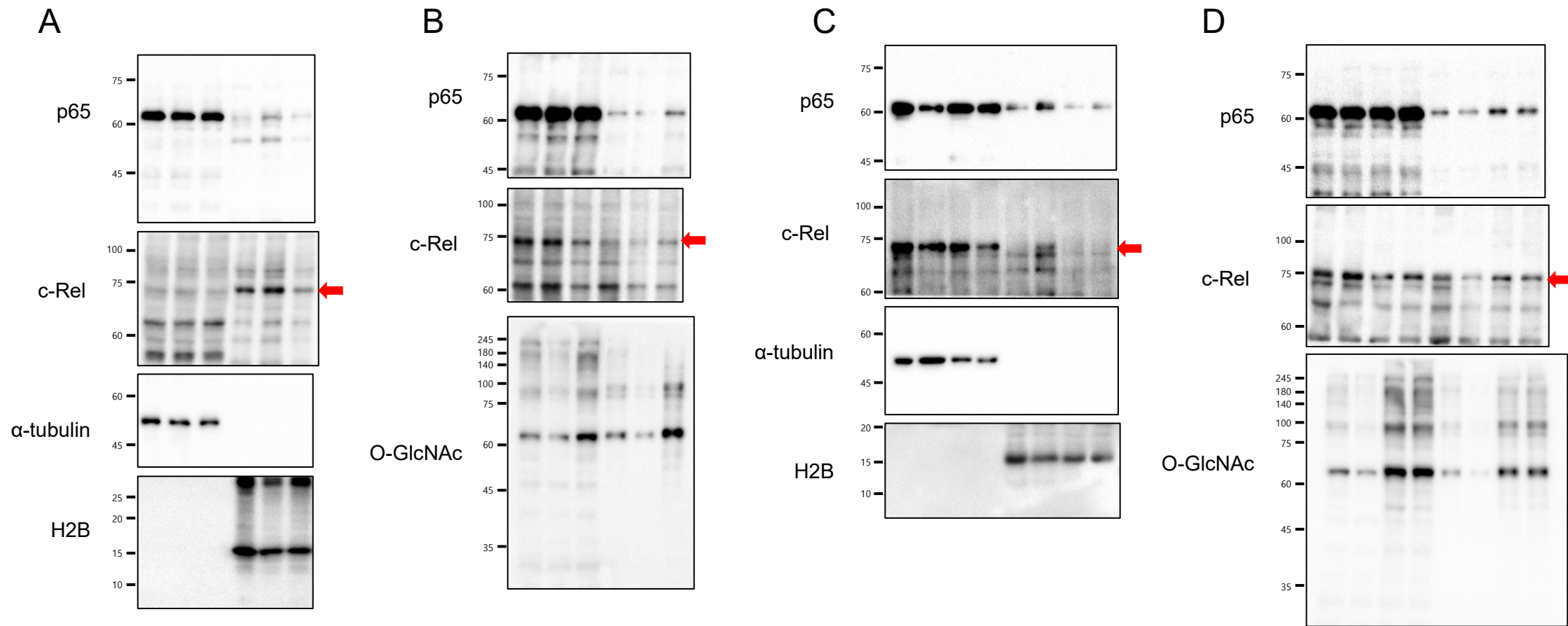

Figure 7

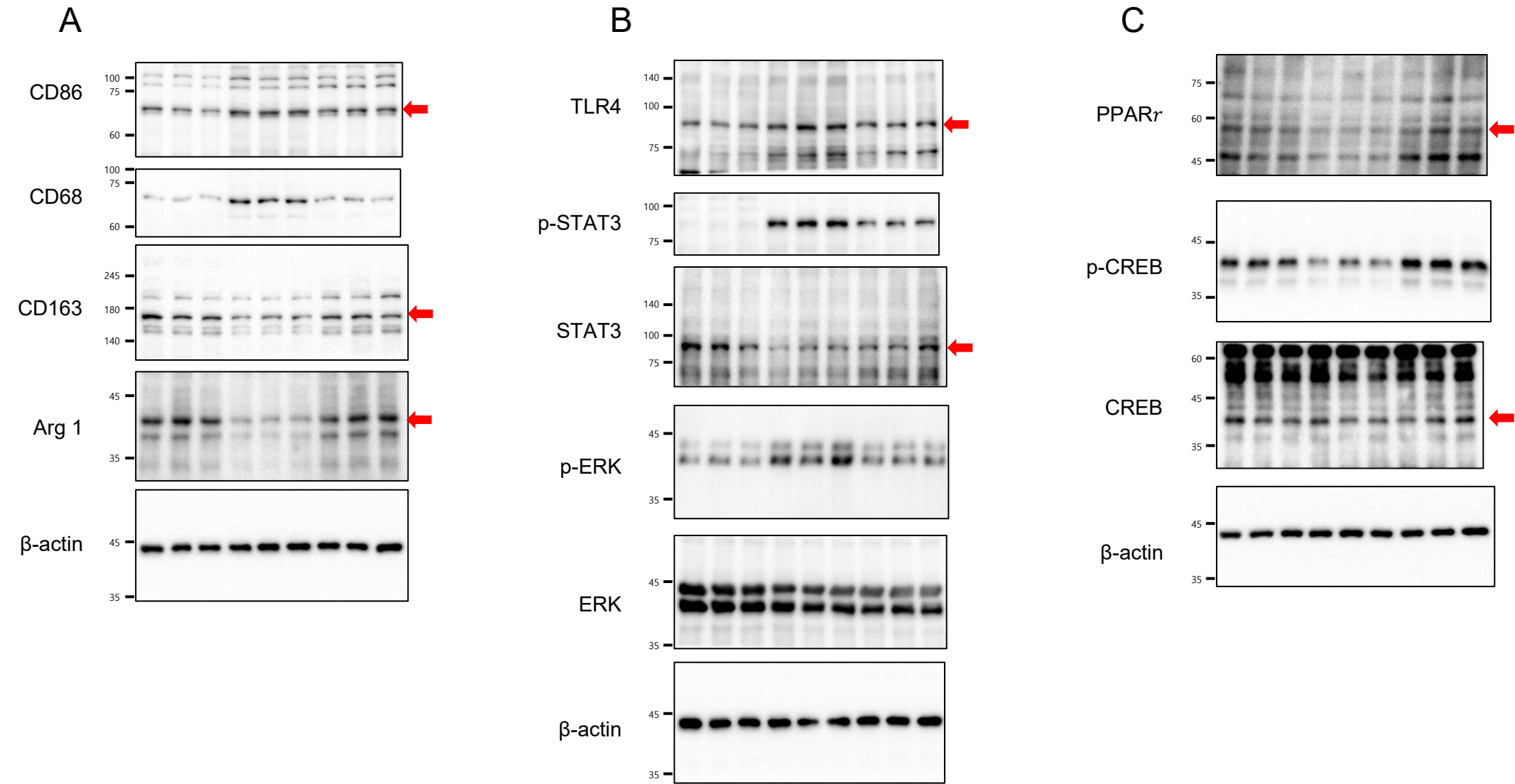

Figure 8

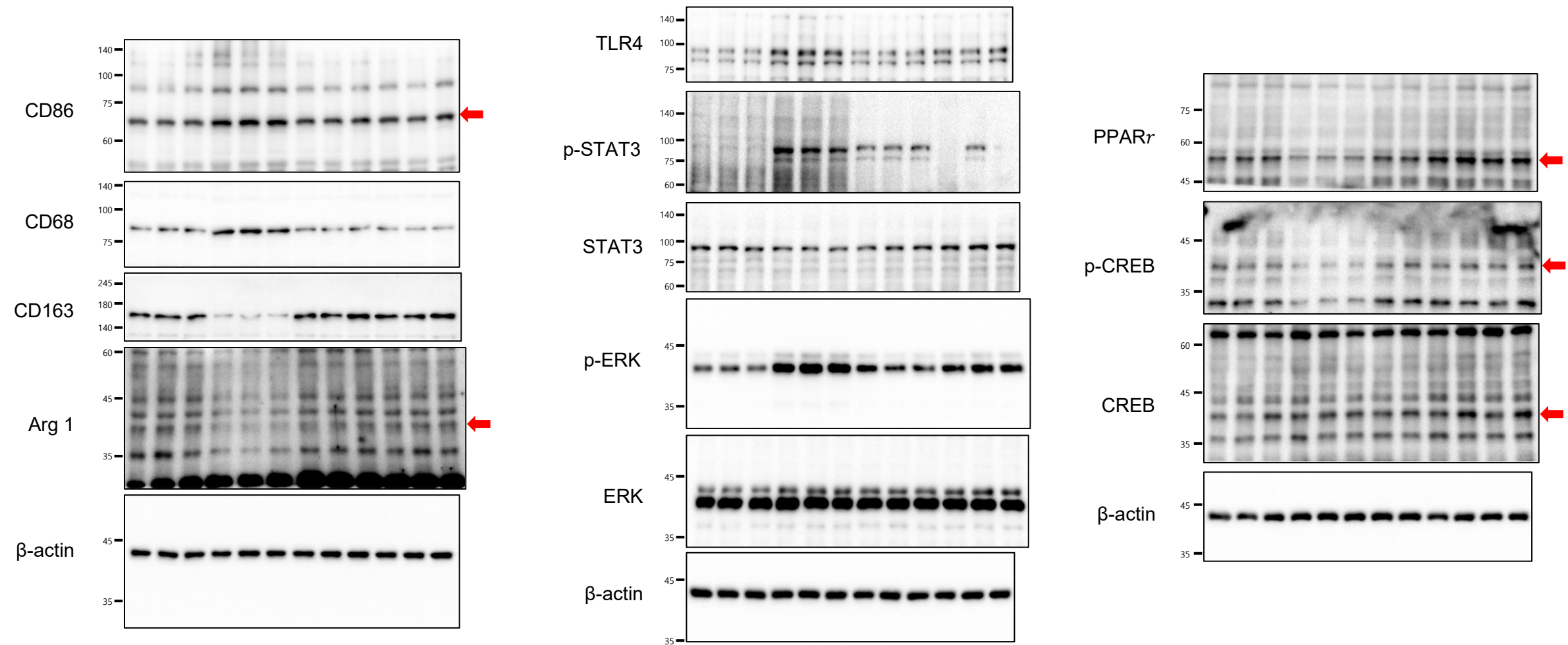

Supplement: Supplementary file 2 — Western bolt original data [file 41419_2026_8862_MOESM2_ESM.pdf]
